# Global burden of young-onset gastric cancer: a systematic trend analysis of the global burden of disease study 2019

**DOI:** 10.1007/s10120-024-01494-6

**Published:** 2024-04-03

**Authors:** Yunhao Li, Anne I. Hahn, Monika Laszkowska, Fang Jiang, Ann G. Zauber, Wai K. Leung

**Affiliations:** 1 Department of Medicine, School of Clinical Medicine, Li Ka Shing Faculty of Medicine, Queen Mary Hospital, The University of Hong Kong, 102 Pokfulam Road, Hong Kong, China; 2 Department of Epidemiology & Biostatistics, Memorial Sloan Kettering Cancer Center, New York, USA; 3 Gastroenterology, Hepatology, and Nutrition Service, Department of Subspecialty Medicine, Memorial Sloan Kettering Cancer Center, New York, USA

**Keywords:** Gastric cancer, Young onset, Global burden, Systematic analysis, Sociodemographic level

## Abstract

**Background:**

While gastric cancer is generally declining globally, the temporal trend of young-onset (< 40 years) gastric cancer remains uncertain. We performed this analysis to determine the temporal trends of young-onset gastric cancer compared to late-onset cancer (≥ 40 years).

**Methods:**

We extracted cross-sectional data from the Global Burden of Diseases, Injuries, and Risk Factors Study (GBD) 2019. The burden of gastric cancer from 1990 to 2019 was assessed through indicators including incidence and mortality rates, which were classified at global, national, and regional levels, and according to socio-demographic indexes (SDI) and age or sex groups. Joinpoint regression analysis was used to identify specific years with significant changes. The correlation between AAPC with countries' average SDI was tested by Pearson’s Test.

**Results:**

The global incidence rate of young-onset gastric cancer decreased from 2.20 (per 100,000) in 1990 to 1.65 in 2019 (AAPC: − 0.95; 95% confidence interval [CI] − 1.25 to − 0.65; *P* < 0.001). Late-onset cancer incidence also decreased from 59.53 (per 100,000) in 1990 to 41.26 in 2019 (AAPC: − 1.23; 95% CI − 1.39 to − 1.06, *P* < 0.001). Despite an overall decreasing trend, the incidence rate of young-onset cancer demonstrated a significant increase from 2015 to 2019 (annual percentage change [APC]: 1.39; 95% CI 0.06 to 2.74; *P* = 0.041), whereas no upward trend was observed in late-onset cancer. Mortality rates of young- and late-onset cancer both exhibited a significant decline during this period (AAPC: − 1.82; 95% CI − 2.15 to − 1.56; *P* < 0.001 and AAPC: − 1.69, 95% CI − 1.79 to − 1.59; P < 0.001). The male-to-female rate ratio for incidence and mortality in both age groups have been increasing since 1990. While countries with high SDI have had a greater decline in the incidence of late-onset gastric cancer (slope of AAPC change: − 0.20, P = 0.004), it was not observed in young-onset cancer (slope of AAPC change: − 0.11, P = 0.13).

**Conclusions:**

The global incidence and mortality rates of both young- and late-onset gastric cancer have decreased since 1990. However, the incidence rate of young-onset cancer has demonstrated a small but significant upward trend since 2015. There was disparity in the decline in young-onset gastric cancer among male and high SDI countries. These findings could help to inform future strategies in preventing gastric cancer in younger individuals.

**Supplementary Information:**

The online version contains supplementary material available at 10.1007/s10120-024-01494-6.

## Introduction

Gastric cancer is a prevalent cancer worldwide, accounting for 5.6% of all new cancer cases (ranking fifth in incidence) and 7.7% of all cancer deaths (ranking fourth in mortality) in 2020 [1]. Gastric cancer is more often diagnosed in individuals over 50 years of age, with younger patients comprising a small proportion and exhibiting relatively low incidence rates [2, 3]. Young-onset gastric cancers often have different clinicopathological characteristics and worse outcomes, including shorter survival rates and lower quality of life, when compared to older populations [4]. Unlike colorectal cancer, there is no universally accepted definition for young-onset gastric cancer, though a cut-off age ranging from 30 to 50 years have been suggested [5–8] and most recent studies have adopted the age of 40 as the cut-off and a definition of 15–39 years was used in studies from the National Cancer Institute of the United States, considering their diverse physiological changes and social transitions [6, 9]. Although the incidence of gastric cancer has gradually decreased worldwide, there remains a lack of understanding regarding the epidemiological trends of young-onset cancer as compared to late-onset cancer [1].

With the increasing use of *Helicobacter pylori (H. pylori)* treatment and improvement in general hygiene and food processing, gastric cancer incidence and mortality rates have steadily declined across most regions of the world since the late twentieth century [10–13]. While a proportion of young-onset gastric cancers are hereditary in nature, additional characteristics and trends driving young-onset cancers remain less explored [14, 15]. Epidemiological data at regional or national levels are particularly limited due to variations in age structure, *H. pylori* infection prevalence, dietary habits, and Universal Health Coverage (UHC) across countries. In a recent cross-sectional study conducted across 48 countries, it was observed that the incidence rate of young-onset gastric cancer increased in several countries such as Ecuador and the UK from 1980 to 2018, but declined in older individuals [16]. However, these findings only reflect a general trend shift observed in a limited number of countries, and it remains unclear whether there have been substantial shifts at specific intervals over this extensive period. In addition, the association between incidence and mortality trends of gastric cancer and the socio-demographic characteristics on national levels remains unknown. Moreover, the potential impact of population-based screening programs on these trends in countries with high gastric cancer incidence, such as Japan and the Republic of Korea, has not been thoroughly considered.

In this study, we aimed to examine the temporal trends of gastric cancer incidence and mortality among individuals with young-onset cancers (< 40 years) at a national and regional level from 1990 to 2019, in comparison to late-onset gastric cancer (≥ 40 years), utilizing data from the Global Burden of Disease (GBD) 2019. We also aimed to identify the periods with the most substantial changes in trends.

## Methods

### Study population and data collection for trend analysis

We retrieved all related cross-sectional data from the Global Health Data Exchange (GHDx) of the GBD 2019 database. This database covers the global burden of 369 diseases in 204 countries or territories over three decades from 1990 to 2019 [17]. Gastric cancer data in GBD 2019 was primarily collected from various sources including medical record review or health facility observation and interviews, etc. Diagnosis of gastric cancer was based on the definition of invasive neoplasms of the stomach, including ICD-10 codes such as C16. Our study adopted the age range of 15–39 years as the operational definition for patients with young-onset gastric cancer and the age range of ≥ 40 years (incorporate age groups: 40–44, 45–49, 50–54, and over 55) for late-onset gastric cancer, similar to prior published studies [18, 19].

Annual data on gastric cancer incidence and mortality were collected from GBD 2019 and stratified based on age, sex, 21 GBD categorized regional groupings of countries (or sub-national administrative regions), six WHO regions, and five socio-demographic index (SDI) levels. The extracted data was used to generate annual male-to-female incidence and mortality rate ratios. The SDI was computed by GBD to reflect the social and economic determinants that may impact individuals' health outcomes across countries. The level of SDI (low, low-middle, middle, high-middle, and high) was determined by the geometric mean of 0 to 1 of the mean number of years of education (among individuals no younger than 15 years), the total fertility rate (among individuals younger than 25 years) and lag distributed income per capita. The cutoff thresholds for countries with low, low-middle, middle, high-middle, and high SDI countries are 0.4547, 0.6076, 0.6895, 0.7905, and 0.8051, respectively [17]. In addition to the aforementioned subgroups, we also extracted data from the three most populous nations in 2019 based on the World Bank population database, namely China, India, and the United States, to determine epidemiological changes in these countries, which may contribute to the major changes in global cancer epidemiology [20]. Since alcohol, smoking and high sodium diet are listed as Group 1 carcinogen by the WHO [21], we have also extracted the annual Summary Exposure Values(SEV) data in GBD 2019 at both global scale and Chinese young individuals (15–39 years) to account for the young-onset gastric cancer in China. SEV is a single, easily interpretable measure that captures the risk-weighted exposure or prevalence of these risk factors for a population. Detailed calculation for SEV has been introduced in previous studies [22].

The incidence rate, mortality rate, incidence cases, and number of deaths were obtained from GBD 2019. The 95% uncertainty intervals (UIs) were defined by the 25th and 75th values of the ordered 1000 estimates based on the GBD's algorithm. Rates were reported per 100,000 population. The detailed methodology used in GBD 2019 has been validated in previous studies [17]. This study follows the Guidelines for Accurate and Transparent Health Estimates Reporting Guidelines for cross-sectional studies [23]. Data acquired was based on the Institute for Health Metrics and Evaluation (IHME's) Free-of-Charge Non-commercial User Agreement.

## Statistical analysis

### Trend analysis

We first examined the global trends of gastric cancer incidence and mortality in young-onset and late-onset patients using age-specific rates and their average annual percentage changes (AAPC). AAPC was calculated by linear regression with rates on the logarithmic scale as the dependent variable and each year as an independent variable. A geometrically weighted average of the annual percentage change (APC) was adopted for calculation in the regression analysis. In this study, we used the AAPC as a singular metric representing the average APC over an extended period, which captured the underlying trend observed from 1990 to 2019. Countries were further classified based on the 25th and 75th percentiles of AAPC and their SDI levels based on the average SDI from 1990 to 2019.

Joinpoint regression analysis was utilized to identify years with notable changes by connecting multiple line segments on a logarithmic scale. The Monte Carlo permutation method was employed for testing when additional joinpoints were introduced [24]. The Weighted Bayesian Information Criterion methods [25] were used to select the final model in the R software (version 4.2.3, package: ljr). Global trends were further stratified by age groups and regional groups. Additional analyses were conducted to examine the temporal trends in male-to-female rate ratios and the proportion of young-onset cancer cases across all age groups. We reported and interpreted the results of statistical tests with effect size and confidence interval (CIs), uncertainty interval, and P values. The Pearson test was used to demonstrate correlations between average SDI (1990–2019) and AAPC of gastric cancer incidence and mortality rates. A P value of less than 0.05 was considered statistically significant.

### Sensitivity analysis

To mitigate potential bias from population screening for gastric cancer, a sensitivity analysis was conducted for the global incidence and mortality rates in the late-onset groups after excluding Japan (which implemented its national screening program in 1983 to people over 40 years) [26] and the Republic of Korea (which implemented its program in 2002) [27]. Additionally, trends of young and late-onset gastric cancer for the two countries were individually analyzed.

Furthermore, to assess the potential impact of different cut-off ages for young-onset gastric cancer, we conducted sensitivity analyses using two additional cut-off ages (< 30 years and < 50 years). Joinpoint analysis of young individuals based on the two different criteria was performed.

## Results

### Global trends of young- and late-onset gastric cancer

The global age-standardized incidence and mortality rate of gastric cancer declined from 22.44 and 20.48 per 100,000 in 1990 to 15.59 and 11.88 in 2019. For young-onset gastric cancer, the global incidence decreased from 2.20 per 100,000 population (95%UI: 2.04 to 2.36) in 1990 to 1.65 per 100,000 (95%UI: 1.52 to 1.79) in 2019, with AAPC of − 0.95 (95% CI − 1.25 to − 0.65, P < 0.001). Rates declined more in females (AAPC: − 1.31; 95% CI − 1.61 to − 1.02, P < 0.001) than males (AAPC: − 0.69; 95%CI − 1.14 to − 0.23, P = 0.003) (Table 1). Mortality rates also decreased from 1.61 per 100,000 population (95%UI: 1.49 to 1.72) in 1990 to 0.94 per 100,000 (95%UI: 0.87 to 1.02) in 2019 (AAPC: − 1.85; 95% CI − 2.15 to − 1.56, P < 0.001) (Table 1). Both sexes demonstrated significant decline in mortality rate over the past 30 years, with females having more pronounced decline than males (AAPC: − 1.99 vs. − 1.63) (Table 1).

**Table 1:** The incidence, mortality, and average annual percentage change (AAPC) of young and late -onset gastric cancer by sex and age from 1990 to 2019

	Incidence						Mortality					
	Case (n), 1990	Incidence (per 100,000), 1990	Case (n), 2019	Incidence (per 100,000), 2019	AAPC (1990–2019)	P value	Case (n), 1990	Mortality (per 100,000), 1990	Case (n),2019	Mortality (per 100,000), 2019	AAPC (1990–2019)	P value
Young-onset												
Global	47,932 (44,593, 51,006)	2.20 (2.04, 2.36)	49,008 (45,008, 53,078)	1.65 (1.52, 1.79)	− 0.95 (− 1.25, − 0.65)	< 0.001	35,270.61 (32,579.02, 37,678.53)	1.61 (1.49, 1.72)	27,896 (25,711, 30,240)	0.94 (0.87, 1.02)	− 1.85 (− 2.15, − 1.56)	< 0.001
Sex												
Male	24,410 (22,660, 26,200)	2.17 (1.95, 2.38)	27,882 (24,956, 31,033)	1.86 (1.66, 2.07)	− 0.69 (− 1.14, − 0.23)	0.003	17,693.57 (16,285.29, 19,068.38)	1.59 (1.47, 1.72)	14,972 (13,643, 16,520)	1.00 (0.91, 1.10)	− 1.63 (− 2.05, − 1.21)	< 0.001
Female	23,521 (21,094, 25,793)	2.19 (2.03, 2.33)	21,126 (18,841, 23,541)	1.44 (1.29, 1.61)	− 1.31 (− 1.61, − 1.02)	< 0.001	17,577.04 (15,561.57, 19,285.91)	1.62 (1.44, 1.78)	12,923 (11,550, 14,339)	0.88 (0.79, 0.98)	− 1.99 (− 2.22, − 1.75)	< 0.001
Late-onset												
Global	834,173.54 (780,579.44, 884,685.66)	59.53 (55.70, 63.13)	1,213,609.87 (1,079,592.51, 1,348,814.09)	41.26 (38.49, 48.08)	− 1.23 (− 1.39 to − 1.06)	< 0.001	750,893.68 (698,388.38, 799,733.52)	53.58 (49.84, 57.07)	919,063.71 (823,742.65, 1,003,818.32)	32.76 (29.37, 35.79)	− 1.69 (− 1.79, − 1.59)	< 0.001
Sex												
Male	523,327.72 (482,330.32, 563,756.00)	76.60 (70.60, 82.51)	816,850.27 (709,624.45, 935,539.83)	59.83 (51.97, 68.52)	− 1.53 (− 1.66, − 1.40)	< 0.001	462,930.08 (423,817.68, 502,497.66)	67.76 (62.03, 73.55)	593,381.48 (521,035.78, 664,049.12)	43.46 (38.16, 48.63)	− 0.86 (− 1.19, − 0.53)	< 0.001
Female	310,845.81 (285,285.79, 333,735.69)	43.28 (39.73, 46.47)	396,759.61 (346,396.07, 444,606.31)	27.56 (24.06, 30.88)	− 1.97 (− 2.09, − 1.85)	< 0.001	287,963.61 (262,615.59, 312,142.16)	40.10 (36.57, 43.47)	325,682.23 (284,322.20, 364,114.71)	22.62 (19.75, 25.29)	− 1.57 (− 1.74, − 1.40)	< 0.001

Data in parentheses are 95% uncertainty intervals for rate and cases for mortality, incidence, and 95% CIs for AAPCs

*UI* uncertainty interval, *CI* confidence interval, *AAPC* average annual percentage change

**Fig. 1:**
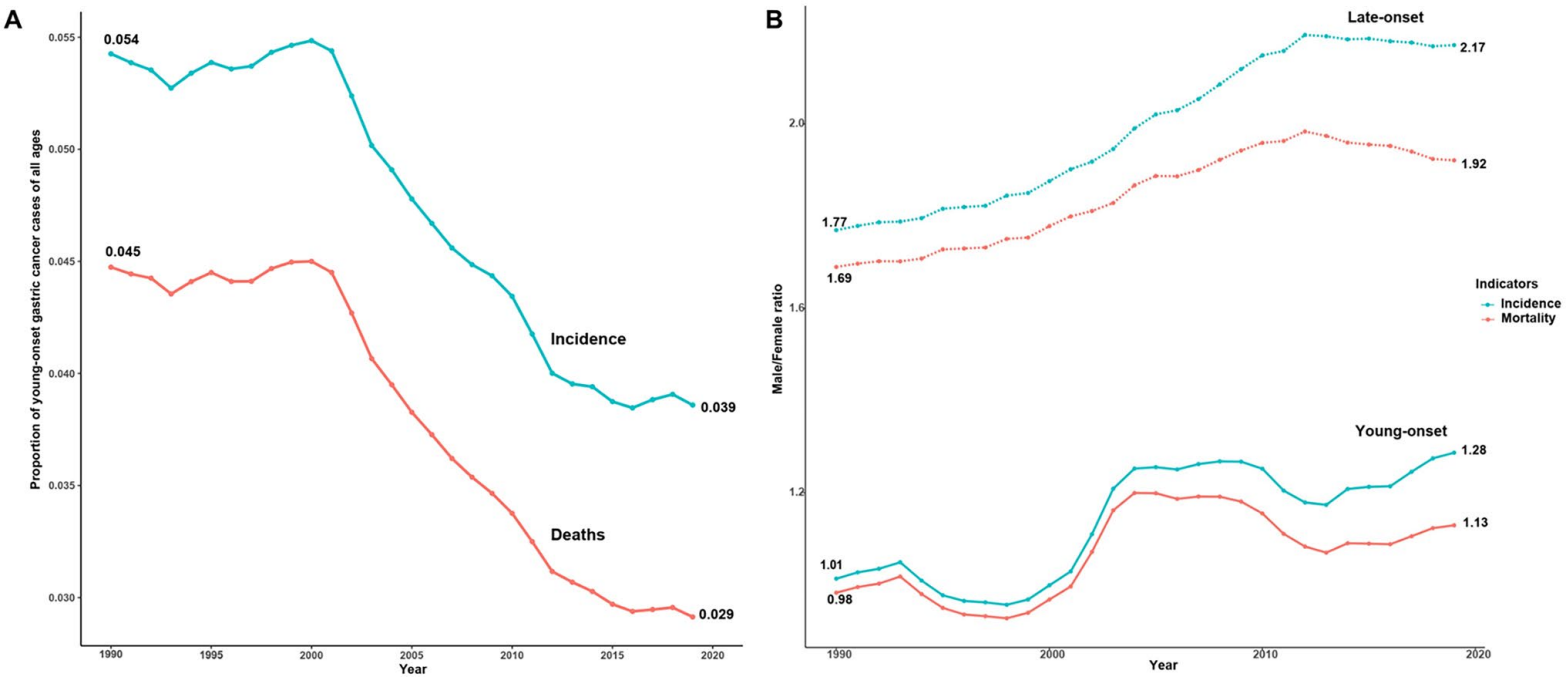
**A** The proportion of young-onset gastric cancer cases and deaths to total cases and deaths. **B** Annual male-to-female ratio change of incidence and mortality rate among young and late-onset gastric cancer

Globally, late-onset gastric cancer incidence also decreased from 59.53 per 100,000 (95% UI: 55.70 to 63.13) in 1990 to 41.26 (95% UI: 38.49 to 48.08) in 2019 (AAPC: − 1.23; 95% CI − 1.39 to − 1.06, P < 0.001). Mortality rates of late-onset gastric cancer decreased from 53.58 per 100,000 (95% UI: 49.84 to 57.07) to 32.76 (95% UI: 29.37 to 35.79) in 2019 (AAPC: − 1.69, 95% CI − 1.79 to − 1.59; P < 0.001) (Table 1). There was a greater percent decline in incidence of late-onset compared to young-onset gastric cancer (30.7% vs. 25.0%, respectively), but not in mortality decline (38.9% vs. 41.6%, respectively). Over the past three decades, there was a general decline in the proportion of young-onset gastric cancer cases to all gastric cancer (Incidence proportion: 0.054 to 0.039; Mortality proportion: 0.045 to 0.029) (Fig. 1A), though this decline has plateaued since 2015.

The male-to-female incidence rate ratio was generally higher in late-onset (range: 1.89–2.30) than young-onset gastric cancer (range: 0.96–1.29). However, due to a more rapid decline in gastric cancer incidence in females, the male predominance has been progressively increasing. The male-to-female incidence rate ratio increased from 1.01 in 1990 to 1.29 in 2019 for young-onset cancer, and from 1.77 in 1990 to 2.17 in 2019 in late-onset cancer. The increasing trends of male-to-female ratio on mortality rate are also noticed in both age groups (Fig. 1B).

**Fig. 2:**
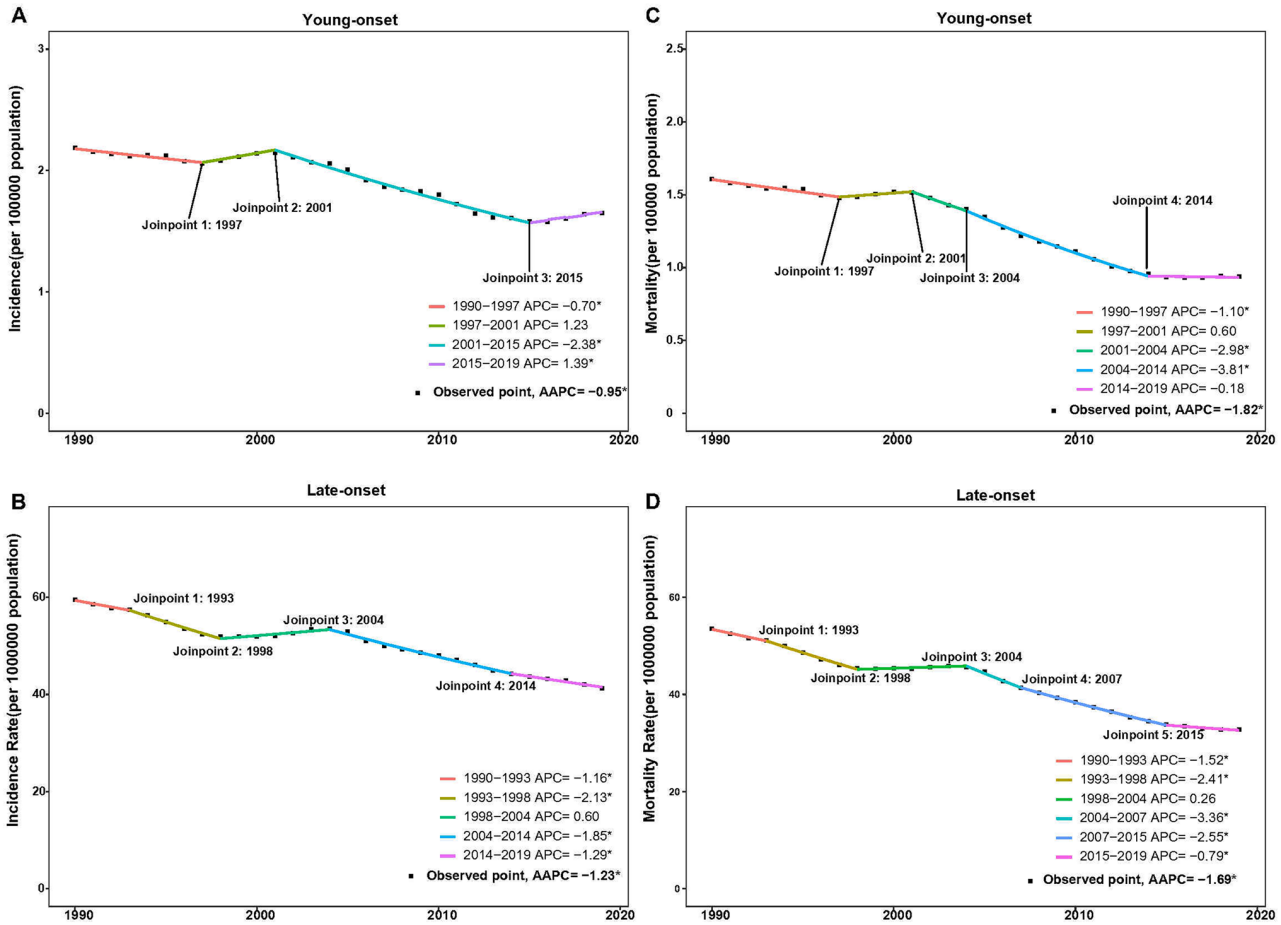
Joinpoint regression analysis comparison of global young-onset and late-onset gastric cancer incidence rate (**A**,**B**), and mortality rate (**C**,**D**), from 1990 to 2019. *APC* annual percentage change, *AAPC* average annual percentage change; *With significance, *P* < 0.05

Joinpoint regression analysis revealed substantial changes in the incidence trends of gastric cancer in 1997, 2002, and 2015. Notably, a significant decline was observed during two periods: from 1990 to 1997 (APC: − 0.70; 95% CI − 1.15 to − 0.26, *P* = 0.004) and from 2002 to 2015 (APC: − 2.38; 95% CI − 2.56 to − 2.20, *P* < 0.001). However, a slight increase in incidence rate of young-onset gastric cancer was observed from 1997 to 2002 (APC:0.60; 95% CI − 2.36 to 2.83; *P* = 0.219) and from 2015 to 2019 (APC: 1.39; 95% CI 0.06 to 2.74; *P* = 0.041; Fig. 2A). There was a consistent decrease in the incidence rate of late-onset cancer since 2004 (2004–2016 APC: − 2.05, 95% CI − 2.18 to − 1.91, P < 0.001; 2014–2019: APC: − 1.29, 95% CI − 1.67 to − 0.92, P < 0.001). A slight increase was noted between 1998 and 2004, but did not reach statistical significance (Fig. 2B).

Notable shifts in mortality rate trends of young-onset gastric cancer were noted in 1997, 2001, 2004, 2013, and 2016 (1990–1997 [APC − 1.08; 95% CI − 1.44 to − 0.72; *P* < 0.001], 2001–2004 [APC: − 2.90; 95% CI − 5.09 to − 0.67; *P* = 0.015], and 2004–2013 [APC: − 3.86; 95% CI − 4.13 to − 3.6; *P* < 0.001], which contributed to the overall decline. The 1997–2001 and 2016–2019 periods exhibited a slight increase in mortality but were not statistically significant (Fig. 2C). Late-onset gastric cancer demonstrated similarly notable joinpoints as the incidence rate trend decreased in 1993, 1998, 2004, 2007, and 2015, with a significant decrease in mortality rate from 2004 (2004–2007 APC: − 3.36, 95% CI − 4.25 to − 2.73, P < 0.001; 2007–2015 APC: − 2.55, 95% CI − 2.67 to − 2.23, P < 0.001; 2015–2019 APC: − 0.79, 95% CI − 0.92 to − 0.67, P < 0.001) (Fig. 2D). Detailed global incidence and mortality rates of young-onset and late-onset gastric cancer by year from 1990 to 2019 are shown in Supplementary Table 1.

### Trend of young- and late-onset gastric cancer by regions and nations

**Fig. 3:**
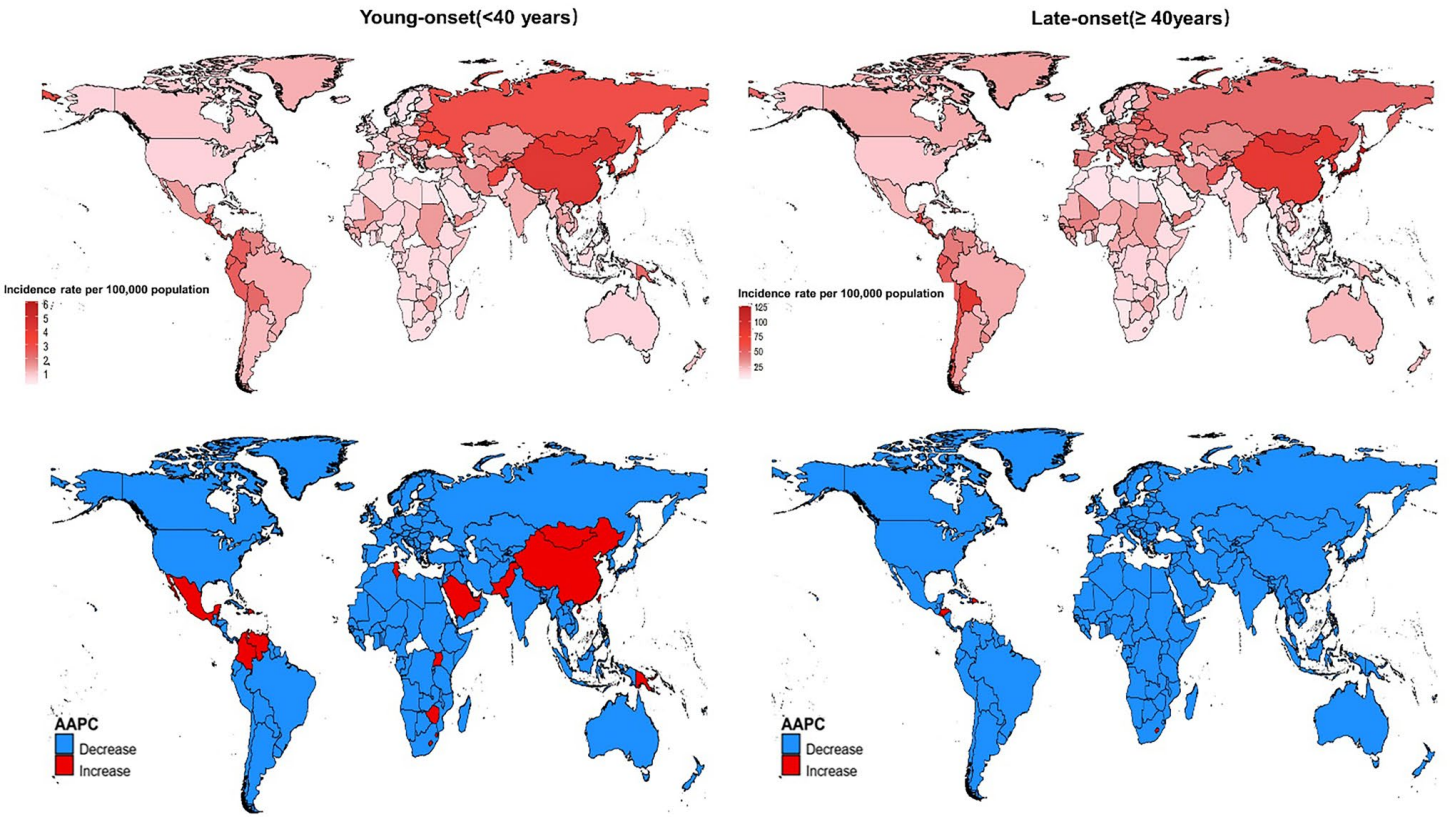
World map of incidence rate in 2019 and AAPC of incidence from 1990 to 2019 for young and late-onset gastric cancer

The global distribution of gastric cancer incidence rates in 2019 and the AAPC for incidence rates from 1990 to 2019 are shown in Fig. 3.

Based on the WHO region classification, the Africa region depicts the steepest decrease of young-onset gastric cancer incidence, from 0.99 per 100,000 population (95% UI: 0.83–1.12) in 1990 to 0.61 (95% UI: 0.51–0.73) in 2019, with AAPC of − 1.67 (95% CI − 1.76 to − 1.59, P < 0.001). However, the Western Pacific region showed a non-significant decrease in incidence (AAPC: − 0.03; 95% CI − 0.52 to 0.48, P = 0.921), while the region of the Americas showed a non-significant increase (AAPC: 0.08; 95% CI − 0.18 to 0.34, P = 0.551; Table 2). Mortality rates of young-onset gastric cancer decreased in all six WHO regions.

**Table 2:** The incidence rate, cases, and average annual percentage change (AAPC) of young and late-onset gastric cancer according to regional stratification from 1990 to 2019

	Incidence-young-onset						Incidence-late-onset					
	Case (n), 1990	Incidence (per 100,000), 1990	Case (n), 2019	Incidence (per 100,000), 2019	AAPC(1990–2019)	P value	Case (n), 1990	Incidence (per 100,000), 1990	Case (n), 2019	Incidence (per 100,000), 2019	AAPC (1990–2019)	P value
WHO regions												
African Region	1902 (1588, 2145)	0.99 (0.83, 1.12)	2705 (2263, 3220)	0.61 (0.51, 0.73)	− 1.67 (− 1.76, − 1.59)	< 0.001	20,660.95 (18,263.79, 22,932.90)	24.16 (21.36, 26.82)	32,741.41 (28,494.89, 37,568.40)	16.25 (14.14, 18.65)	− 1.35 (− 1.43, − 1.27)	< 0.001
Eastern Mediterranean Region	1462 (1214, 1669)	1.02 (0.84, 1.16)	2924 (2481, 3430)	0.96 (0.81, 1.13)	− 0.16 (− 0.23, − 0.1)	< 0.001	17,649.50 (14,687.10, 19,858.82)	25.34 (21.09, 28.51)	34,790.02 (30,537.49, 39,055.42)	19.64 (17.24, 22.04)	− 0.84 (− 0.95, − 0.79)	< 0.001
European Region	7879 (7571, 8123)	2.40 (2.31, 2.48)	4767 (4391, 5183)	1.56 (1.44, 1.70)	− 1.45 (− 1.95, − 0.94)	< 0.001	219,010.15 (210,377.60, 225,142.42)	65.35 (62.78, 67.18)	177,397.19 (158,737.75, 194,883.22)	38.62 (34.56, 42.43)	− 1.76 (− 2.10, − 1.42)	< 0.001
Region of the Americas	3898 (3774, 4001)	1.33 (1.29, 1.37)	5033 (4446, 5731)	1.33 (1.17, 1.51)	0.08 (− 0.18, 0.34)	0.551	74,514.04 (70,886.31, 76,984.22)	36.68 (34.89, 37.89)	113,206.17 (99,502.73, 126,930.00)	27.96 (24.58, 31.36)	− 0.92 (− 1.13, − 0.70)	< 0.001
South-East Asia Region	8112 (6934, 8947)	1.54 (1.31, 1.69)	9528 (8391, 10,769)	1.12 (0.99, 1.27)	− 1.05 (− 1.32, − 0.79)	< 0.001	67,648.33 (60,600.02, 74,942.46)	24.35 (21.82, 26.98)	113,527.85 (99,776.01, 128,691.87)	17.87 (15.70, 20.25)	− 1.06 (− 1.25, − 0.87)	< 0.001
Western Pacific Region	24,584 (22,182, 27,252)	3.50 (3.16, 3.88)	23,961 (20,725, 27,602)	3.52 (3.04, 4.05)	− 0.03 (− 0.52, 0.48)	0.921	432,935.66 (389,528.54, 478,330.49)	101.67 (91.48, 112.34)	739,412.30 (625,308.58, 862,416.21)	80.57 (68.14, 93.98)	− 0.87 (− 1.12, − 0.55)	< 0.001
GBD regions												
Andean Latin America	452 (400, 508)	2.92 (2.59, 3.29)	633 (486, 799)	2.47 (1.89, 3.11)	− 0.55 (− 1.18, 0.08)	0.088	5503.55 (4801.21, 6251.18)	71.75 (62.59, 81.49)	11,624.50 (9059.66, 14,597.76)	58.75 (45.79, 73.78)	− 0.80 (− 1.33, − 0.27)	0.003
Australasia	75 (68, 83)	0.92 (0.83, 1.01)	74 (56, 95)	0.76 (0.58, 0.98)	− 0.50 (− 1.23, 0.1)	0.096	2297.98 (2077.99, 2519.39)	30.54 (27.61, 33.48)	3330.38 (2479.08, 4345.47)	24.12 (17.95, 31.47)	− 0.81 (− 1.16, − 0.46)	< 0.001
Caribbean	169 (136, 191)	1.14 (0.92, 1.29)	197 (153, 242)	1.09 (0.84, 1.33)	− 0.10 (− 0.4, 0.15)	0.376	2748.19 (2444.63, 3036.01)	30.47 (27.11, 33.66)	4119.17 (3416.73, 4887.51)	23.81 (19.75, 28.25)	− 0.77 (− 1.09, − 0.46)	< 0.001
Central Asia	1009 (963, 1061)	3.54 (3.38, 3.73)	744 (664, 838)	1.96 (1.75, 2.21)	− 2.00 (− 2.46, − 1.55)	< 0.001	12,360.49 (11,666.89, 13,033.23)	78.15 (73.77, 82.41)	11,373.38 (10,023.03, 12,899.24)	39.59 (34.89, 44.90)	− 2.32 (− 2.47, − 2.17)	< 0.001
Central Europe	810 (782, 841)	1.76 (1.70, 1.83)	363 (315, 418)	1.02 (0.89, 1.17)	− 1.87 (− 2.12, − 1.61)	< 0.001	25,618.99 (24,505.96, 26,598.68)	53.40 (51.08, 55.44)	21,270.58 (18,372.23, 24,262.27)	34.96 (30.19, 39.87)	− 1.47 (− 1.70, − 1.24)	< 0.001
Central Latin America	1193 (1150, 1234)	1.75 (1.69, 1.81)	1938 (1604, 2315)	1.92 (1.59, 2.29)	0.3 (0.11, 0.63)	0.005	14,256.80 (13,434.55, 14,944.94)	44.85 (42.26, 47.01)	28,332.16 (23,541.13, 33,713.15)	33.95 (28.21, 40.40)	− 1.04 (− 1.34, − 0.73)	< 0.001
Central Sub-Saharan Africa	230 (166, 293)	1.11 (0.80, 1.41)	342 (250, 439)	0.66 (0.48, 0.85)	− 1.8 (− 1.91, − 1.68)	< 0.001	2493.84 (1913.82, 3204.12)	27.95 (21.45, 35.90)	3904.06 (2905.41, 5153.88)	17.21 (12.81, 22.72)	− 1.65 (− 1.82, − 1.48)	< 0.001
East Asia	18,729 (16,352, 21,292)	3.30 (2.88, 3.76)	21,313 (18,107, 24,881)	4.13 (3.51, 4.82)	0.70 (0.26, 1.32)	0.003	306,849.68 (266,099.64, 351,019.05)	94.96 (82.35, 108.62)	604,309.35 (501,696.73, 719,636.03)	83.55 (69.36, 99.49)	− 0.48 (− 0.76, − 0.20)	0.001
Eastern Europe	3516 (3256, 3669)	4.10 (3.80, 4.28)	2037 (1794, 2303)	2.97 (2.61, 3.36)	− 1.10 (− 2.28, 0.09)	0.07	83,458.23 (79,675.02, 86,458.07)	93.57 (89.33, 96.93)	51,965.70 (46,196.12, 58,301.04)	49.91 (44.37, 55.99)	− 2.01 (− 2.91, − 1.11)	< 0.001
Eastern Sub-Saharan Africa	823 (603, 969)	1.17 (0.86, 1.37)	1109 (908, 1387)	0.67 (0.54, 0.83)	− 1.92 (− 2.06, − 1.77)	< 0.001	7406.93 (6319.74, 8442.60)	25.29 (21.58, 28.82)	10,628.01 (9098.27, 12,338.04)	15.53 (13.29, 18.02)	− 1.68 (− 1.78, − 1.58)	< 0.001
High-income Asia Pacific	5218 (5006, 5429)	7.72 (7.41, 8.04)	1767 (1550, 2024)	3.36 (2.95, 3.85)	− 2.84 (− 3.37, − 2.32)	< 0.001	118,342.64 (111,674.50, 123,953.74)	167.46 (158.03, 175.40)	123,423.22 (100,650.21, 144,524.93)	111.53 (90.95, 130.60)	− 1.42 (− 1.78, − 1.06)	< 0.001
High-income North America	878 (846, 910)	0.78 (0.75, 0.80)	915 (791, 1064)	0.75 (0.65, 0.88)	− 0.04 (− 0.5, 0.43)	0.870	29,239.50 (27,526.74, 30,494.00)	27.55 (25.93, 28.73)	36,108.54 (30,755.62, 41,639.68)	20.53 (17.48, 23.67)	− 1.01 (− 1.22, − 0.79)	< 0.001
North Africa and Middle East	1919 (1634, 2196)	1.41 (1.20, 1.62)	2770 (2341, 3277)	1.07 (0.91, 1.27)	− 0.93 (− 1.03, − 0.83)	< 0.001	37,649.18 (35,617.80, 39,203.63)	45.32 (42.87, 47.19)	67,368.91 (58,971.52, 76,096.52)	32.84 (28.75, 37.10)	− 1.29 (− 1.38, − 1.19)	< 0.001
Oceania	61 (46, 76)	2.31 (1.76, 2.89)	134 (98, 178)	2.46 (1.80, 3.26)	0.17 (0.06, 0.29)	0.003	21,532.30 (18,152.26, 24,409.36)	32.88 (27.72, 37.27)	39,355.93 (34,419.40, 44,493.67)	22.60 (19.77, 25.55)	− 0.38 (− 0.52, − 0.23)	< 0.001
South Asia	6322 (5438, 7037)	1.46 (1.26, 1.63)	8655 (7508, 9913)	1.13 (0.98, 1.29)	− 0.89 (− 1.21, − 0.57)	< 0.001	358.65 (270.34, 466.15)	29.66 (22.36, 38.55)	802.48 (578.85, 1086.69)	26.72 (19.27, 36.18)	− 0.87 (− 1.22, − 0.51)	< 0.001
Southeast Asia	2593 (2128, 2953)	1.32 (1.08, 1.50)	2242 (1924, 2634)	0.83 (0.71, 0.97)	− 1.58 (− 1.7, − 1.46)	< 0.001	51,235.48 (45,152.94, 57,601.17)	22.61 (19.92, 25.41)	90,633.04 (78,001.57, 104,926.30)	17.45 (15.02, 20.20)	− 1.63 (− 1.75, − 1.51)	< 0.001
Southern Latin America	284 (266, 301)	1.49 (1.39, 1.58)	301 (227, 388)	1.18 (0.89, 1.53)	− 0.80 (− 1.2, − 0.39)	< 0.001	25,433.40 (21,676.42, 28,980.39)	26.03 (22.19, 29.66)	37,684.47 (32,369.66, 43,476.21)	16.16 (13.88, 18.64)	− 1.03 (− 1.38, − 0.67)	< 0.001
Southern Sub-Saharan Africa	254 (228, 278)	1.16 (1.04, 1.27)	250 (199, 310)	0.74 (0.59, 0.92)	− 1.48 (− 2.23, − 0.72)	< 0.001	8147.47 (7541.76, 8765.04)	52.53 (48.62, 56.51)	10,307.27 (7875.36, 13,290.81)	39.12 (29.89, 50.45)	− 1.07 (− 1.52, − 0.61)	< 0.001
Tropical Latin America	942 (901, 982)	1.47 (1.40, 1.53)	1061 (998, 1130)	1.19 (1.12, 1.27)	− 0.74 (− 1.01, − 0.47)	< 0.001	2158.85 (1887.97, 2439.26)	21.20 (18.54, 23.95)	3351.49 (2938.11, 3818.24)	15.76 (13.82, 17.96)	− 1.55 (− 1.73, − 1.38)	< 0.001
Western Europe	1914 (1852, 1980)	1.33 (1.29, 1.37)	1178 (1010, 1367)	0.90 (0.77, 1.04)	− 1.42 (− 1.69, − 1.15)	< 0.001	15,140.64 (14,217.80, 15,987.54)	43.76 (41.09, 46.20)	23,293.07 (21,258.13, 25,129.24)	27.54 (25.13, 29.71)	− 1.42 (− 1.59, − 1.26)	< 0.001
Western Sub-Saharan Africa	543 (453, 626)	0.76 (0.64, 0.88)	982 (791, 1197)	0.55 (0.44, 0.67)	− 1.12 (− 1.24, − 1.01)	< 0.001	91,615.69 (86,727.97, 95,331.41)	54.14 (51.25, 56.34)	83,849.95 (70,908.83, 96,349.21)	35.62 (30.13, 40.93)	− 0.99 (− 1.06, − 0.92)	< 0.001
SDI level												
Low SDI	2450 (1959,2810)	1.26 (1.01,1.45)	3845 (3250,4409)	0.86 (0.73,0.98)	− 1.32 (− 1.45, − 1.19)	< 0.001	24,303.67 (21,193.65, 27,268.27)	26.33 (22.97, 29.55)	39,280.53 (34,598.28, 44,260.67)	18.89 (16.63, 21.28)	− 1.15 (− 1.20, − 1.11)	< 0.001
Low-middle SDI	7869 (6880,8641)	1.77 (1.54,1.94)	10,020 (8965,11,051)	1.36 (1.22,1.50)	− 0.88 (− 1.12, − 0.64)	< 0.001	77,868.54 (70,482.13, 85,387.07)	33.73 (30.53, 36.99)	138,877.86 (124,615.87, 153,927.45)	27.51 (24.69, 30.49)	− 0.71 (− 0.81, − 0.61)	< 0.001
Middle SDI	16,800 (15,186,18,427)	2.25 (2.03,2.47)	17,805 (15,817,20,003)	1.90 (1.69,2.14)	− 0.5 (− 0.94, − 0.06)	0.025	234,410.82 (208,323.92, 263,176.83)	60.31 (53.60, 67.72)	439,498.54 (377,927.40, 508,052.32)	48.45 (41.66, 56.01)	− 0.76 (− 0.92, − 0.60)	< 0.001
High-middle SDI	12,913 (11,980,13,927)	2.67 (2.48,2.88)	13,171 (11,760,14,732)	2.55 (2.28,2.85)	− 0.1 (− 0.64, 0.37)	0.586	278,371.11 (259,580.76, 296,974.94)	76.65 (71.48, 81.77)	366,574.16 (318,156.94, 418,780.55)	54.92 (47.67, 62.75)	− 1.15 (− 1.39, − 0.91)	< 0.001
High SDI	7886 (7656,8114)	2.44 (2.37,2.51)	4147 (3824,4510)	1.25 (1.15,1.36)	− 2.33 (− 2.61, − 2.04)	< 0.001	218,976.94 (208,315.62, 226,605.31)	67.23 (63.96, 69.57)	229,029.10 (197,997.87, 255,491.57)	44.36 (38.35, 49.48)	− 1.41 (− 1.70, − 1.13)	< 0.001

Data in parentheses are 95% uncertainty intervals for rate and cases for mortality, incidence, and 95% CIs for AAPCs

*UI* uncertainty interval, *CI* confidence interval, *AAPC* average annual percentage change, *SDI* socio-demographic index

In contrast, a significant decrease in both incidence and mortality rates were observed across all WHO regions for late-onset gastric cancer. The European region demonstrated a significant decrease in both incidence (AAPC: − 1.76; 95% CI − 2.10 to − 1.42, P < 0.001) and mortality rate (AAPC: − 2.22; 95% CI − 2.55 to − 1.88, P < 0.001) (Table 2 and Supplementary Table 2), while the Eastern Mediterranean Region exhibited the least decline in incidence (AAPC: − 0.84; 95% CI − 0.95 to − 0.79, P < 0.001) and mortality rates (AAPC: − 1.04; 95% CI − 1.11 to − 0.97, P < 0.001) over the past three decades.

When different GBD regions were examined, most regions reported a decreasing incidence of young- or late-onset gastric cancer (Table 2). However, Central Latin America (AAPC: 0.30; 95% CI 0.11–0.63, P = 0.005), East Asia (AAPC: 0.70; 95% CI 0.26–1.32, P = 0.030), and Oceania (AAPC: 0.17; 95% CI 0.06–0.29, P = 0.003) reported a significant increase in young-onset gastric cancer incidence rate. Moreover, an increase in mortality rate (AAPC: 0.18; 95% CI 0.08–0.29, P = 0.001) was observed in young-onset gastric cancer in Oceania (Supplementary Table 2).

When stratified by individual nations, Cyprus (AAPC: 2.02; 95% CI 1.14–2.91, P < 0.001) and Lesotho (AAPC: 1.92; 95% CI 1.36–2.49, P < 0.001) exhibited the most significant rise in the incidence rates of young-onset cancer (Table 3). At the same time, Lesotho (AAPC: 1.89; 95% CI 1.59–2.19, P < 0.001) and Zimbabwe (AAPC: 1.62; 95% CI 0.93–2.33, P < 0.001) were found to have the largest increase in mortality rates of young-onset cancer (Supplementary Table 3). Cyprus and Lesotho, the two countries with the most significant increases in young-onset gastric cancer incidence rates, also exhibited a significantly increase in incidence rate of late-onset cancer (AAPC: 0.42, P = 0.004 and AAPC: 0.34, P < 0.001 respectively) (Table 3). As for late-onset cancer, Dominican Republic demonstrated the most increase in incidence rate (AAPC: 1.07; 95% CI 0.12–2.03, P = 0.028). United States Virgin Islands observed the largest increase in mortality rate (AAPC: 0.98; 95% CI 0.71 to 1.25, P < 0.001) (Supplementary Table 3). Andorra, Cyprus, Guam, Australia, San Marino, and Canada were countries (or subnational administrative area) with high SDI but low-level AAPC in both young- and late-onset cancer. In contrast, Ethiopia, and Rwanda were countries with low SDI but high AAPC for young- and late-onset gastric cancer (Supplementary Fig. 1). A detailed list of the countries with significant increased incidence and mortality rates is shown in Table 3 and Supplementary Table 3.

**Table 3:** List of countries with significantly increased average annual percentage changes of young and late-onset gastric cancer incidence rate

Country	WHO regions	GBD-region	GBD-Super region	AAPC (95% confidence interval)	P value
Young-onset					
Eswatini	African Region	Southern Sub‐Saharan Africa	Sub‐Saharan Africa	0.54 (0.03, 1.05)	0.038
Lesotho	African Region	Southern Sub‐Saharan Africa	Sub‐Saharan Africa	1.92 (1.36, 2.49)	< 0.001
Zimbabwe	African Region	Southern Sub‐Saharan Africa	Sub‐Saharan Africa	1.47 (0.94, 2.01)	< 0.001
Lebanon	Eastern Mediterranean Region	North Africa and Middle East	North Africa and Middle East	0.28 (0.05, 0.51)	0.017
Pakistan	Eastern Mediterranean Region	South Asia	South Asia	0.50 (0.29, 0.72)	< 0.001
Saudi Arabia	Eastern Mediterranean Region	North Africa and Middle East	North Africa and Middle East	1.09 (0.83, 1.36)	< 0.001
Tunisia	Eastern Mediterranean Region	North Africa and Middle East	North Africa and Middle East	0.29 (0.10, 0.48)	0.003
United Arab Emirates	Eastern Mediterranean Region	North Africa and Middle East	North Africa and Middle East	0.59 (-0.01, 1.20)	0.055
Andorra	European Region	Western Europe	High income	0.25 (0.08, 0.42)	0.004
Cyprus	European Region	Western Europe	High income	2.02 (1.14, 2.91)	< 0.001
Belize	Region of the Americas	Caribbean	Latin America and Caribbean	1.15 (0.37, 1.93)	0.004
Dominican Republic	Region of the Americas	Caribbean	Latin America and Caribbean	0.80 (0.31, 1.29)	0.001
Mexico	Region of the Americas	Central Latin America	Latin America and Caribbean	0.68 (0.07, 1.28)	0.028
China	Western Pacific Region	East Asia	Southeast Asia, East Asia, and Oceania	0.84 (0.3, 1.39)	0.002
Guam	Western Pacific Region	Oceania	Southeast Asia, East Asia, and Oceania	1.33 (0.49, 2.18)	0.002
Marshall Islands	Western Pacific Region	Oceania	Southeast Asia, East Asia, and Oceania	0.52 (0.44, 0.59)	< 0.001
Samoa	Western Pacific Region	Oceania	Southeast Asia, East Asia, and Oceania	0.08 (0.02, 0.15)	0.013
Solomon Islands	Western Pacific Region	Oceania	Southeast Asia, East Asia, and Oceania	0.57 (0.36, 0.79)	< 0.001
Tonga	Western Pacific Region	Oceania	Southeast Asia, East Asia, and Oceania	0.47 (0.23, 0.71)	< 0.001
Vanuatu	Western Pacific Region	Oceania	Southeast Asia, East Asia, and Oceania	0.46 (0.24, 0.69)	< 0.001
Late-onset					
Cyprus	European Region	Western Europe	High income	0.42 (0.13, 0.70)	0.004
El Salvador	Region of the Americas	Caribbean	Latin America and Caribbean	0.66 (0.11, 1.22)	0.018
Honduras	Region of the Americas	Central Latin America	Latin America and Caribbean	0.74 (0.36, 1.13)	< 0.001
Lesotho	African Region	Southern Sub‐Saharan Africa	Southern Sub‐Saharan Africa	0.34 (0.22, 0.45)	< 0.001
United States Virgin Islands	Region of the Americas	High‐income North America	High‐income	1.20 (0.89, 1.50)	< 0.001

*AAPC* average annual percentage change

**Fig. 4:**
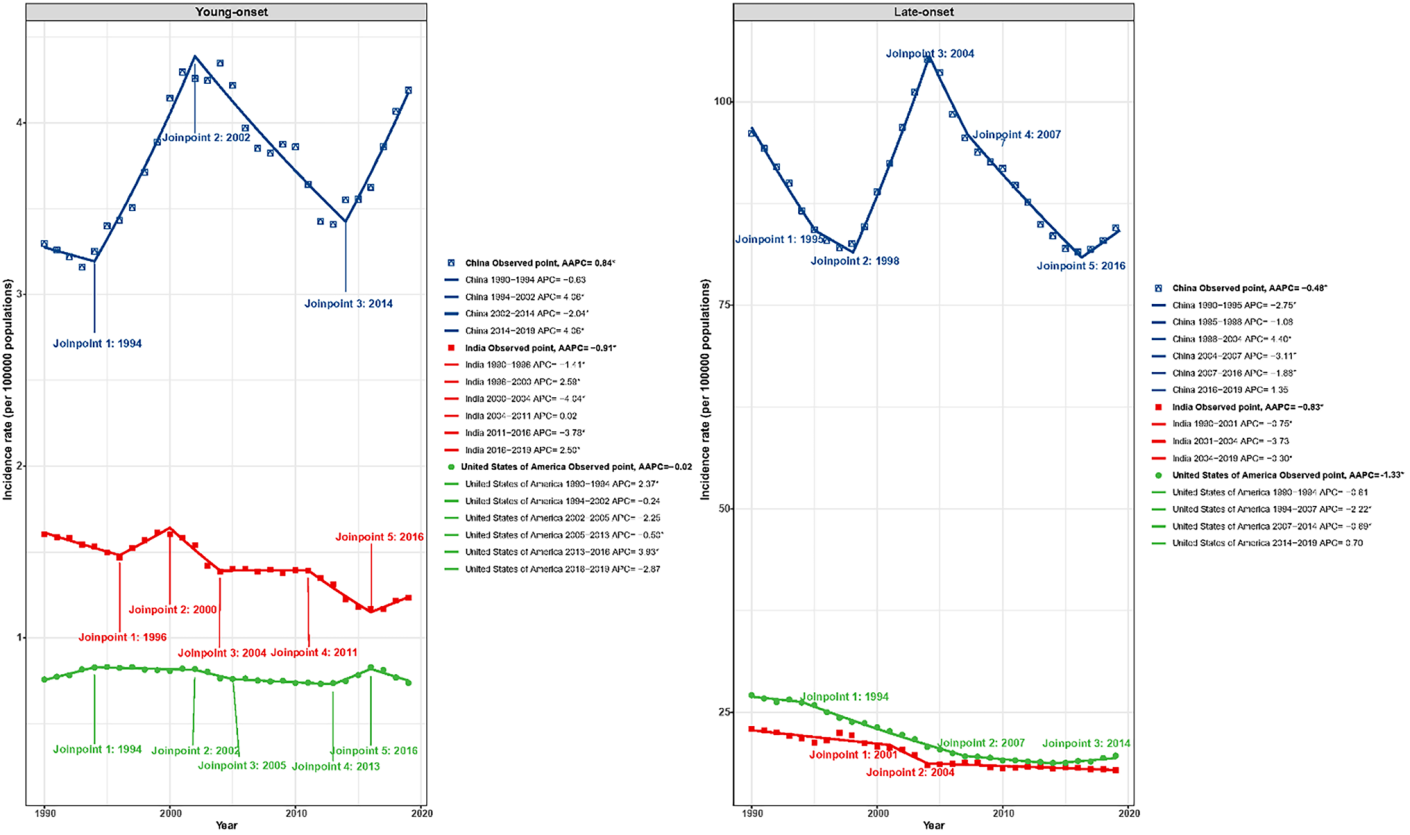
Joinpoint regression analysis comparison of China, India, and the United States of America for young and late-onset gastric cancer incidence rate. *APC* annual percentage change, *AAPC* average annual percentage change; *With significance, *P* < 0.05

China, with the world's largest population during the study period, was the only East Asia country that showed a significant increase in the incidence rate of young-onset gastric cancer over the study period (AAPC: 0.84; 95% CI 0.30–1.39, P = 0.002) according to either GBD or UNSD [28] geographical region criteria (Supplementary Fig. 2). Furthermore, the Joinpoint analysis results among the three most populous countries (China, India and the United States of America) indicate a significant increase in young-onset gastric cancer incidence rates in China after 2014 (APC: 4.06, 95% CI 2.00–6.15, P = 0.001) and in India after 2016 (APC: 2.50, 95% CI 0.47–4.58, P = 0.020), respectively. However, no similar trend was observed for late-onset gastric cancer in these countries (Fig. 4). The fluctuations in the incidence and mortality rate of young-onset gastric cancer in the U.S. remained relatively stable over past three decades. Among the three most populous countries, the U.S. exhibited the slowest decline in incidence (AAPC: − 0.02, 95% CI − 0.55 to 0.51, P = 0.947), but the most rapid decline in mortality (AAPC: − 1.13, 95% CI − 1.35 to − 0.91, P < 0.001) (Fig. 4).

In terms of the exposure to carcinogens associated with young-onset gastric cancer in China, it was found that young individuals from China had significantly higher levels of smoking exposure (SEV: 8.50–9.68) compared to the global average (6.34–9.50). For high sodium diets, despite a decline in the global average SEV from 49.39 in 1990 to 43.27 in 2019, young individuals in China have consistently maintained a relatively stable level of exposure to high-sodium diets (94.97–96.70). Moreover, alcohol consumption in China experienced a significant increase from 2005 and has exceeded the global average since 2012 (Supplementary Fig. 3).

### Sensitivity analysis

Japan and the Republic of Korea, both countries with a significant burden of gastric cancer, have implemented national screening programs for individuals over 40 years old. Both countries exhibited an overall decline in incidence and mortality rates from 1990 to 2019 for both young and late-onset gastric cancers. For late-onset cancer, the decline in incidence (AAPC: − 2.05 vs. − 1.21) and mortality (AAPC: − 3.82 vs. − 0.92) was more pronounced in the Republic of Korea than in Japan. Moreover, there has been a significant increase in both incidence (2016–2019 APC: 2.40, 95% CI 0.46–4.37, P = 0.018) and mortality rates (2016–2019 APC: 95% CI 0.82, 95% CI 0.15–1.49, P = 0.021) in Republic of Korea after 2016 for late-onset cancer, which was not observed in Japan (Supplementary Fig. 4A). For young-onset gastric cancer in these two countries, the Republic of Korea had higher incidence and mortality rates than Japan. While Japan experienced a greater decline in incidence rates (AAPC: − 3.44 vs. − 2.32), the reduction in mortality was steeper in the Republic of Korea (AAPC: − 4.03 vs. − 5.01). Similar to late-onset cancer, there was a non-significant increase in young onset gastric cancer incidence observed in Republic of Korea after 2016 (APC: 3.43, 95% CI − 2.12 to 9.30, *P* = 0.213) (Supplementary Fig. 4B). Also, when compared the ratio of young-onset gastric cancer incidence rates in the Republic of Korea and Japan with global incidence rates, it was found that the burden of gastric cancer in the Republic of Korea remains high globally, with an increasing trend observed after 2016 (from 2.72 to 2.78) (Supplementary Fig. 5).

After excluding data from these two countries with universal screening, the decline in global incidence rate (AAPC: − 0.99, 95% CI − 1.15 to − 0.83, P < 0.001) and mortality rate (AAPC: − 1.68, 95% CI − 1.78 to − 1.58, P < 0.001) of late-onset gastric cancer was still observed but at a lower magnitude (AAPC: − 1.23 and − 1.69 respectively) (Supplementary Fig. 6).

When considering different cutoff ages for young-onset gastric cancer, a significant decline in the incidence and mortality rates of young gastric cancer was still observed between 1990 and 2019 with cutoff values of 30 or 50 (All *P* value for AAPC < 0.05). However, there has been a consistent decrease in both incidence and mortality since 2010 with these cut-offs, instead of the observed increase in incidence rate from 2015 with cut-off age of 40 years. (Supplementary Figs. 7, 8).

### Trend of young- and late-onset gastric cancer according to SDI

According to the SDI quintiles, the most significant decrease in incidence rate was observed in the high SDI quintile, both for young-onset (AAPC: − 2.33, 95% CI − 2.61 to − 2.04, P < 0.001) and late-onset gastric cancer (AAPC: − 1.53, 95% CI − 1.33 to − 1.73, P < 0.001) (Table 2). For both age groups, the mortality rate decreased significantly in all five SDI quintiles (Supplementary Table 2).

**Fig. 5:**
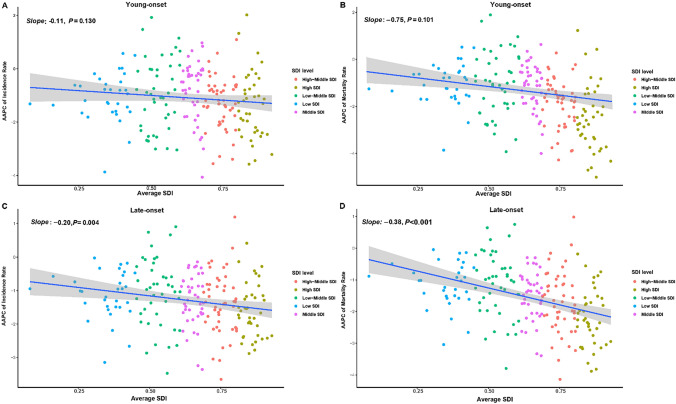
Correlation of average SDI with AAPC of incidence and mortality rate in young and late-onset gastric cancer. *AAPC* average annual percentage change, *SDI* Sociodemographic Index

The incidence and mortality rates of young-onset gastric cancer did not exhibit a significant decline with increasing SDI (AAPC of incidence rate Slope: − 0.11, P = 0.13; AAPC of mortality rate, Slope: − 0.75, P = 0.101) (Fig. 5A and B). However, there was a significant decline in both the AAPC of incidence rate (Slope: − 0.20, P = 0.004; Fig. 5C) and mortality rate (Slope: − 0.38, P < 0.001; Fig. 5D) of late-onset gastric cancer with increase in the country’s SDI. Higher SDI countries had a more rapid decline of incidence or mortality rate of late-onset gastric cancer from 1990 to 2019.

## Discussion

In this study, we have demonstrated the most updated global trends of the incidence and mortality of young-onset as compared to late-onset gastric cancer, including regional, sex-based, and SDI-based estimates. Although there has been a general decline in the incidences of both young- and late-onset gastric cancer, there was a small but significant increase in incidence rates of young-onset gastric cancer in the period of 2015 to 2019. Moreover, while there was a decline detected in most WHO or GBD regions, some GBD regions still showed a significant increase in the incidences of young-onset gastric cancer, including Central Latin America, East Asia, and Oceania. The decline was more rapid in females than males. There was also marked heterogeneity between individual nations and countries with different SDIs. Although countries with high SDI generally exhibited a greater magnitude of decline in incidence rates of late-onset gastric cancer, this trend was not apparent for young-onset gastric cancer.

Notably, we observed a marked difference in the trends of gastric cancer incidence and mortality at regional and national levels. Central Latin America, East Asia, and Oceania are three GBD regions that have had an increase in gastric cancer incidence rates among young individuals. The reasons for this remain unclear. In Latin America, gastric cancer is still one of the most common cancers, and countries including Costa Rica and Colombia reported the highest mortality rate of gastric cancer globally [29]. In our study, Central Latin America countries including Mexico, Colombia, and Venezuela showed an increase in incidence rates. Torres et al. suggested that the geographical disparities in gastric cancer incidence across the Americas may stem from factors such as altitude, host genetics, genotypes, prevalence of *H. pylori* infection and dietary habits [29]. Effective campaigns targeting *H. pylori* eradication in Latin America have yet to be implemented, which may also contribute to the persistently high rates of gastric cancer [30]. On the other hand, China is the only country with a significant increase in incidence rate of young-onset cancer in East Asia. The increasing incidence of gastric cancer in Chinese young adults may be attributed to the high background rate of *H. pylori* infection and lack of a universal screening program for gastric cancer [31, 32]. It is also evident that younger individuals from China had significantly higher levels of exposure to smoking and high-salt diets when compared to the global average. Furthermore, alcohol consumption in China has surpassed the global average since 2012 (Supplementary Fig. 3). Together, these may partly explain the heavy burden of young-onset gastric cancer in China and the significant increase in recent years, which could also be attributed to increased exposure to these unhealthy lifestyle habits associated with gastric cancer. Despite an overall declining trend in the burden of gastric cancer among young individuals in Africa, some low-income countries such as Lesotho and Zimbab still exhibit an increase in both incidence and mortality rates in younger individuals. This may be related to the low universal health coverage in these populations and lack of screening for gastric cancer and *H. pylori* infection [33, 34].

Joinpoint regression model results indicate an overall decreasing young-onset and late-onset gastric cancer incidence and mortality rate from 1990 to 2019. However, a small but significant increase in incidence rate was observed after 2015 for young-onset gastric cancer. The underlying reasons for the increase are unclear. This may be partly due to the increasing use of screening and surveillance procedures among younger individuals, with the detection of more cancers at earlier stages. Multiple guidelines from the North America and Europe have been established since 2015 to underscore the importance of monitoring for gastric preneoplastic lesions like chronic atrophic gastritis, intestinal metaplasia, and dysplasia, which may enhance public awareness regarding the significance of surveillance and hence early cancer prevention [35–37]. Moreover, as the Joinpoint analysis result shows, the changes in incidence in populous countries, such as mainland China and India, may also contribute to the increase due to the large burden of cases in these countries [38]. The United Nations predicts that India will surpass China as the world's most populous country in 2027 [39]. Therefore, it is imperative to increase the awareness of the escalating incidence of young-onset gastric cancer in India, despite its relatively low background incidence rate. The overall prevalence of *H. pylori* has decreases in the United States in recent decades, but Andersen et al. identified an increase in the incidence of non-cardia gastric cancer among young (< 50 years) U.S. non-Hispanic white females from 1995 to 2013. This finding may be attributed to the long lag time between *H. pylori* infection and gastric cancer development which may not be apparent within a relatively short period, or may potentially be related to the rise of other risk factors such as autoimmune gastritis in this population [40]. Our research group has recently reported a consistent declining prevalence of *H. pylori* infection globally [41]. However, there is limited data available regarding temporal changes in *H. pylori* infection prevalence rates among younger individuals and their association with changes in gastric cancer incidence. To gain insights into its potential association, further large-scale epidemiological studies are needed.

Socioeconomic barriers or inequalities frequently prevent individuals from getting early detection and medical care, affecting cancer prevention and treatment outcomes. This study found the largest decrease in late-onset gastric cancer incidence rates occurred in countries with high SDI. Furthermore, the correlation between AAPC of incidence or mortality rate with countries’ SDI indicated that a higher sociodemographic level was associated with a more rapid decline of disease burden, particularly evident in late-onset gastric cancer. On the other hand, similar association between SDI and the rate of decline of young-onset gastric cancer is not apparent, which suggest additional interventions may be needed to curb the rising incidence of gastric cancer in some high-risk countries. The incidence of *H. pylori* infection is also closely linked to a country's sociodemographic status as well as universal healthcare coverage [42, 43]. Low SDI countries usually have higher prevalence of *H. pylori* infection and suboptimal implementation of other related measures, ultimately leading to a higher incidence of gastric cancer [44, 45]. This study suggests that the rate of decline in gastric cancer incidence and mortality is largely driven by countries with high SDI levels rather than low SDI levels, and more resources are needed to address these needs in low SDI countries.

Japan and the Republic of Korea, being countries with high incidence of gastric cancer, have implemented screening programs for individuals aged 40 and above. Radiographic screening programs for gastric cancer have been proactively implemented from the 1960s. In 1999, the Republic of Korea introduced a biennial national endoscopic screening program, resulting in a 47% reduction in mortality from gastric cancer compared to no screening [46]. While gastric cancer screening program has been implemented in Japan and the Republic of Korea for individuals aged 40 and above, younger individuals are not eligible in the gastric cancer program. Hence, the decline in gastric cancer incidence among young individuals is likely attributed to other factors such as *H. pylori* eradication, opportunistic screening and improvement in other socioeconomic factors.

Based on the results of our study, we observed a more rapid decline in gastric cancer incidences in female than male, for both young- and late-onset gastric cancers. While differences in the temporal trends of gastric cancer incidence between male and female were widely studied, the underlying reasons are still under debate [47]. Further efforts on the promotion of healthy lifestyle and screening or surveillance may be needed to combat gastric cancer in high-risk regions. These could be attributed to difference in genetic, environmental and lifestyle factors (such as smoking and drinking habits) that contribute to the disparity in decline in gastric cancer incidence between male and female. Furthermore, past studies suggested the protective effects of exposure to estrogen on gastric cancer, which might contribute to a more rapid decrease of incidence among females. With the globally declining prevalence of smoking, this may have an even more positive impact on late-onset gastric cancer.

There are several limitations of this study. Although the data in this study were obtained from GBD 2019, an authoritative burden of disease database, it may not fully reflect the epidemiological characteristics of the disease. Data scarcity has always been the primary challenge faced by GBD [48]. Under circumstances when data is unavailable, some countries utilize comparable national data or employ a similar spatial model for smoothing [49] or utilize spline models to fit missing years. Other databases, such as the WHO's Global Health Estimates, might curate relevant data from multiple registries [50]. It is important to note that after excluding the Republic of Korea and Japan, we failed to observe a significant increase in incidence rates after 2015. This could be attributed to the substantial load of gastric cancer cases from the Republic of Korea which could impact on the global trend. Specifically, the incidence rate in the Republic of Korea exhibited a significant upward trend compared to the global incidence rate during this period (Supplementary Fig. 5). It is also imperative to acknowledge the potential biases arising from statistical noise [51] and underreporting of gastric cancer cases in other countries. Second, it is essential to consider other socio-demographic factors, such as race, health coverage, income level, educational level, lifestyle risk factors and even the prevalence of *H. pylori* infection in the evaluation process, as we solely utilize the SDI index level as an independent variable to represent a country's socio-demographic status. Third, to ensure a more homogeneous study population for our trend analysis, the main results were mainly based on the cut-off age of 40 years. However, sensitivity analyses with different cut-off ages of 30 and 50 years have been performed. Fourth, another major limitation of our study is the lack of results pertaining to cardia versus non-cardia gastric cancer, due to data unavailability within the GBD database. Several regional studies have indicated the disparities of clinicopathological characteristics and epidemiological features between cardia and non-cardia cancer [52–54]. Lastly, clinical or pathological staging data, which determine the prognosis and therapy, are also unavailable. Therefore, further large-scale global epidemiological studies are eagerly needed in the future.

## Conclusion

Overall, global gastric cancer incidence and mortality rates have shown a progressive decline from 1990, particularly in countries with high socio-demographic levels. However, an increase in incidence of young-onset gastric cancer is notable in some countries and regions since 2015, which could be driven by the increase in some densely populated regions. Females have a more rapid decline in gastric cancer incidences for both young- and late-onset gastric cancers. In general, countries with lower SDIs exhibited a slower decline in gastric cancer incidence and mortality, particularly for late-onset but less apparent in young-onset cancers. Our findings could help to inform future strategies for early detection of young-onset gastric cancer, highlighting areas and regions where early detection, treatment of *H. pylori* infection, lifestyle modification and judicious use of screening procedures may be necessary to curb the rising trend.

### Supplementary Information

Below is the link to the electronic supplementary material.

Supplementary file1 (DOCX 1899 KB)

## Data Availability

Data generated or analyzed in this article are included in the supplementary material.
