# Supplementary material for: Global burden of young-onset gastric cancer: a systematic trend analysis of the global burden of disease study 2019
Source: Gastric Cancer. 2024 Apr 3;27(4):684–700. doi: 10.1007/s10120-024-01494-6 (PMC11193827; doi:10.1007/s10120-024-01494-6)
Supplement: Supplementary file 1 — Supplementary file1 (DOCX 1899 KB) [file 10120_2024_1494_MOESM1_ESM.docx]

**ORIGINAL STUDY-Supplementary Material**

**GLOBAL BURDEN OF YOUNG-ONSET GASTRIC CANCER: A SYSTEMATIC TREND ANALYSIS OF THE GLOBAL BURDEN OF DISEASE STUDY 2019**

**Content List:**

**Supp Table 1:** The incidence and mortality rates of young-onset gastric cancer from 1990 to 2019 at the global level………………………**Page 2-8**

**Supp Table 2:** The mortality rate, cases, and average annual percentage change (AAPC) of young and late-onset gastric cancer according to regional stratification from 1990 to 2019…………………………………………………………………………………………………………………………….**Page 9-17**

**Supp Table 3:** List of countries with significantly increased average annual percentage changes of mortality rate………………………………**Page 18-20**

**Supp Figure 1:** Countries classification based on AAPC level (25 and 75 percentile) and SDI level……………………………………………..**Page 21**

**Supp Figure 2:** Joinpoint regression result of incidence rate in East Asia(UNSD classification)………………………………………………….**Page 22**

**Supp Figure 3:** Summary Exposure Value of alcohol use, smoking, and High-sodium Diet of Global and China among young individual……**Page 23**

**Supp Figure 4:** Joinpoint regression result of Japan and the Republic of Korea and Japan………………………………………………………**Page 24-25**

**Supp Figure 5:** Temporal trend of young-onset gastric cancer incidence rate ratio between the Republic of Korea & Japan versus the global level…………………………………………………………………………………………………………………………………………………………..**Page 26**

**Supp Figure 6:** Joinpoint regression result of global data excluding Japan and the Republic of Korea………………………………………….**Page 27**

**Supp Figure 7:** Joinpoint regression result of young gastric cancer incidence and Mortality (cut-off age at 30 years)……………………………………………………………………………………………………………………………………………….……….**Page 28**

**Supp Figure 8:** Joinpoint regression result of young gastric cancer incidence and Mortality (cut-off age at 50 years)……………………………………………………………………………………………………………………………………………….……….**Page 29**

**Supplementary Table 1** The incidence and mortality rates of young-onset gastric cancer from 1990 to 2019 at the global level.

| **Year** | **Incidence(UI)** | | | **Mortality(UI)** | | **Year** | **Incidence(UI)** | | **Mortality(UI)** | |
| --- | --- | --- | --- | --- | --- | --- | --- | --- | --- | --- |
|  | | **case** | **Rate** | **case** | **Rate** |  | **case** | **Rate** | **case** | **Rate** |
| **1990** | | 47932(44593,51006) | 2.19(2.03, 2.33) | 35271(32579,37679) | 1.61(1.49, 1.72) | **2005** | 53908(51426,57153) | 2.01(1.92, 2.13) | 36213(34427,38370) | 1.35(1.28, 1.43) |
| **1991** | | 47966(44986,50880) | 2.16(2.02, 2.29) | 35154(32629,37476) | 1.58(1.47, 1.68) | **2006** | 52066(49642,55315) | 1.92(1.83, 2.04) | 34569(32834,36724) | 1.28(1.21, 1.36) |
| **1992** | | 48161(45261,50979) | 2.14(2.01, 2.26) | 35243(33002,37537) | 1.56(1.46, 1.67) | **2007** | 51090(48923,54479) | 1.87(1.79, 1.99) | 33326(31849,35468) | 1.22(1.16, 1.30) |
| **1993** | | 48311(45757,50898) | 2.12(2.00, 2.23) | 35194(33006,37221) | 1.54(1.45, 1.63) | **2008** | 50912(48683,53999) | 1.84(1.76, 1.96) | 32593(31126,34745) | 1.18(1.13, 1.26) |
| **1994** | | 49123(46823,51616) | 2.13(2.03, 2.23) | 35748(33819,37678) | 1.55(1.46, 1.63) | **2009** | 50864(48670,53944) | 1.83(1.75, 1.94) | 31885(30532,33771) | 1.15(1.10, 1.21) |
| **1995** | | 49659(47274,52009) | 2.12(2.02, 2.22) | 35998(34077,37832) | 1.54(1.46, 1.62) | **2010** | 50452(48044,53695) | 1.80(1.72, 1.92) | 31140(29740,33062) | 1.11(1.06, 1.18) |
| **1996** | | 49293(46888,51683) | 2.08(1.98, 2.18) | 35520(33737,37319) | 1.50(1.42, 1.57) | **2011** | 48608(46367,51844) | 1.73(1.65, 1.84) | 29840(28461,31776) | 1.06(1.01, 1.13) |
| **1997** | | 49520(47174,51767) | 2.06(1.96, 2.15) | 35506(33629,37237) | 1.48(1.40, 1.55) | **2012** | 46642(44456,49818) | 1.65(1.57, 1.76) | 28530(27207,30381) | 1.01(0.96, 1.07) |
| **1998** | | 50796(47847,53304) | 2.08(1.96, 2.19) | 36261(34128,38031) | 1.49(1.40, 1.56) | **2013** | 46033(43748,49136) | 1.61(1.53, 1.72) | 27909(26613,29549) | 0.98(0.93, 1.04) |
| **1999** | | 52261(49455,54827) | 2.11(2.00, 2.22) | 37229(35073,39029) | 1.50(1.42, 1.58) | **2014** | 46111(43777,49039) | 1.61(1.53, 1.71) | 27452(26078,29022) | 0.96(0.91, 1.01) |
| **2000** | | 53782(51021,56462) | 2.14(2.03, 2.25) | 38214(36059,40194) | 1.52(1.44, 1.60) | **2015** | 45705(43171,48770) | 1.58(1.49, 1.69) | 27058(25624,28679) | 0.94(0.89, 0.99) |
| **2001** | | 54653(51952,57573) | 2.14(2.04, 2.26) | 38635(36601,40698) | 1.52(1.44, 1.60) | **2016** | 45865(43191,48982) | 1.58(1.49, 1.68) | 27046(25564,28750) | 0.93(0.88, 0.99) |
| **2002** | | 54507(51732,57156) | 2.11(2.00, 2.21) | 38222(36212,40122) | 1.48(1.40, 1.55) | **2017** | 46971(43819,50439) | 1.60(1.50, 1.72) | 27274(25350,29177) | 0.93(0.87, 1.00) |
| **2003** | | 54191(51841,56773) | 2.07(1.98, 2.17) | 37442(35587,39420) | 1.43(1.36, 1.50) | **2018** | 48383(44749,52209) | 1.64(1.52, 1.77) | 27747(25664,29974) | 0.94(0.87, 1.02) |
| **2004** | | 54579(52099,57660) | 2.06(1.96, 2.17) | 37145(35425,39306) | 1.40(1.34, 1.48) | **2019** | 49008(45008,53078) | 1.65(1.52, 1.79) | 27895(25711,30240) | 0.94(0.87, 1.02) |

Note: UI: uncertainty interval;

**Supplementary Table 1(Continued)**. The incidence and mortality rates of late-onset gastric cancer from 1990 to 2019 at the global level.

| **Year** | **Incidence(UI)** | | | **Mortality(UI)** | | **Year** | **Incidence(UI)** | | **Mortality(UI)** | |
| --- | --- | --- | --- | --- | --- | --- | --- | --- | --- | --- |
|  | | **case** | **Rate** | **case** | **Rate** |  | **case** | **Rate** | **case** | **Rate** |
| **1990** | | 834173.54(780579.44, 884685.66) | 59.53(55.70, 63.13) | 750893.68(698388.38, 799733.52) | 53.58(49.84, 57.07) | **2005** | 1070797.63(1003139.59, 1133177.44) | 52.94(49.59, 56.02) | 904645.79(848440.73, 957041.50) | 44.72(41.95, 47.31) |
| **1991** | | 841185.18(788324.18, 892253.44) | 58.57(54.89, 62.13) | 753665.90(704606.72, 803291.31) | 52.48(49.06, 55.94) | **2006** | 1059323.69(993215.18, 1116721.49) | 51.00(47.82, 53.76) | 887340.34(829725.53, 936928.66) | 42.72(39.95, 45.11) |
| **1992** | | 850023.73(798325.55, 901384.10) | 57.78(54.26, 61.27) | 758806.86(709492.70, 806354.57) | 51.58(48.23, 54.81) | **2007** | 1065400.47(997351.44, 1129339.51) | 49.96(46.77, 52.96) | 881125.00(824046.30, 929752.28) | 41.32(38.64, 43.60) |
| **1993** | | 866393.72(814772.50, 913406.25) | 57.48(54.05, 60.60) | 770441.67(723366.95, 816859.34) | 51.11(47.99, 54.19) | **2008** | 1079986.08(1009965.73, 1142607.09) | 49.34(46.15, 52.21) | 882587.70(823586.03, 932095.53) | 40.33(37.63, 42.59) |
| **1994** | | 869290.20(822880.44, 918011.87) | 56.27(53.26, 59.42) | 772303.92(725642.50, 818193.61) | 49.99(46.97, 52.96) | **2009** | 1091446.96(1016285.19, 1156660.93) | 48.61(45.27, 51.52) | 881749.46(822433.80, 931207.01) | 39.27(36.63, 41.48) |
| **1995** | | 870422.74(824544.31, 914805.64) | 54.96(52.06, 57.76) | 770106.28(726881.37, 814546.31) | 48.63(45.90, 51.43) | **2010** | 1106408.36(1026501.71, 1174858.24) | 48.06(44.59, 51.03) | 884473.69(822519.12, 936471.33) | 38.42(35.73, 40.67) |
| **1996** | | 868777.51(823700.27, 914346.70) | 53.54(50.76, 56.34) | 766871.00(723835.57, 809888.51) | 47.26(44.60, 49.91) | **2011** | 1110665.52(1029887.66, 1181712.34) | 47.06(43.64, 50.07) | 881335.94(816466.53, 937844.60) | 37.34(34.59, 39.74) |
| **1997** | | 870534.86(825084.31, 915823.02) | 52.40(49.66, 55.12) | 766206.74(724239.28, 807687.22) | 46.12(43.59, 48.61) | **2012** | 1114176.38(1032071.07, 1187119.64) | 46.08(42.69, 49.10) | 879611.36(816507.84, 935131.65) | 36.38(33.77, 38.68) |
| **1998** | | 882077.84(834877.86, 927459.78) | 51.90(49.12, 54.57) | 771869.38(728389.43, 813736.31) | 45.41(42.85, 47.87) | **2013** | 1113259.46(1027419.79, 1193040.15) | 44.98(41.51, 48.20) | 874017.60(806661.25, 930076.36) | 35.31(32.59, 37.58) |
| **1999** | | 901923.28(853307.63, 946004.99) | 51.88(49.08, 54.42) | 786959.12(741630.38, 829639.01) | 45.27(42.66, 47.72) | **2014** | 1118671.31(1029271.14, 1197328.45) | 44.19(40.66, 47.30) | 871607.80(803472.42, 930932.40) | 34.43(31.74, 36.77) |
| **2000** | | 924348.04(874473.92, 972541.56) | 51.96(49.16, 54.67) | 807042.62(760869.01, 852569.75) | 45.37(42.77, 47.93) | **2015** | 1128289.45(1032523.26, 1213518.25) | 43.61(39.91, 46.91) | 875623.85(804871.43, 935711.29) | 33.85(31.11, 36.17) |
| **2001** | | 947610.67(890849.92, 1000059.01) | 52.02(48.90, 54.89) | 825248.12(774764.47, 872415.96) | 45.30(42.53, 47.89) | **2016** | 1140496.21(1037571.32, 1231318.29) | 43.17(39.28, 46.61) | 884684.41(808001.12, 954042.45) | 33.49(30.59, 36.11) |
| **2002** | | 983244.76(924500.17, 1035412.84) | 52.65(49.50, 55.44) | 852544.05(799685.51, 901948.22) | 45.65(42.82, 48.30) | **2017** | 1156199.07(1042704.91, 1259627.09) | 42.89(38.68, 46.72) | 889084.10(807764.45, 959190.52) | 32.98(29.96, 35.58) |
| **2003** | | 1022944.98(961696.68, 1081134.11) | 53.37(50.17, 56.40) | 878582.70(824833.07, 927889.71) | 45.83(43.03, 48.41) | **2018** | 1183187.73(1057416.38, 1299018.36) | 42.02(38.45, 47.23) | 901205.34(812070.02, 984894.80) | 32.77(29.53, 35.81) |
| **2004** | | 1054109.09(988447.43, 1117749.88) | 53.54(50.20, 56.77) | 898265.99(842966.25, 949856.18) | 45.62(42.81, 48.24) | **2019** | 1213609.87(1079592.51, 1348814.09) | 41.26(38.49, 48.08) | 919063.71(823742.65, 1003818.32) | 32.76(29.37, 35.79) |

**Supplementary Table 2** The mortality rate, cases, and average annual percentage change (AAPC) of young and late-onset gastric cancer according to regional stratification from 1990 to 2019

|  | **Mortality-young-onset** |  |  |  |  |  | **Mortality-late-onset** |  |  |  |  |  |
| --- | --- | --- | --- | --- | --- | --- | --- | --- | --- | --- | --- | --- |
|  | **case(n),1990** | **Mortality(per 100000),1990** | **case(n),2019** | **Mortality(per 100000),2019** | **AAPC(1990-2019)** | **P value** | **case(n),1990** | **Mortality(per 100000),1990** | **case(n),2019** | **Mortality(per 100000),2019** | **AAPC(1990-2019)** | **P value** |
| **WHO regions** | | | | | | | | | | | | |
| African Region | 1557(1301, 1759) | 0.81(0.68, 0.92) | 2225(1843, 2654) | 0.50(0.42, 0.60) | -1.62(-1.75 , -1.5) | <0.001 | 21554.29(19072.03, 23929.06) | 25.20(22.30, 27.98) | 34191.45(29700.15, 39034.56) | 16.97(14.74, 19.38) | -1.35(-1.43, -1.27) | <0.001 |
| Eastern Mediterranean Region | 1183(981, 1349) | 0.82(0.68, 0.94) | 2226(1867, 2647) | 0.73(0.61, 0.87) | -0.4(-0.47 , -0.32) | <0.001 | 18185.31(15135.20, 20461.60) | 26.11(21.73, 29.38) | 34053.46(29860.12, 38272.33) | 19.22(16.85, 21.60) | -1.04(-1.11, -0.97) | <0.001 |
| European Region | 5636(5402, 5826) | 1.72(1.65, 1.78) | 2752(2543, 2970) | 0.90(0.83, 0.97) | -2.18(-2.66 , -1.7) | <0.001 | 201223.31(192902.99, 206927.70) | 60.05(57.56, 61.75) | 142919.47(129685.18, 153219.52) | 31.11(28.23, 33.36) | -2.22(-2.55, -1.88) | <0.001 |
| Region of the Americas | 2818(2725, 2900) | 0.96(0.93, 0.99) | 3168(2839, 3545) | 0.84(0.75, 0.94) | -0.43(-0.65 , -0.2) | <0.001 | 66838.94(63369.74, 69148.02) | 32.90(31.19, 34.04) | 93386.29(83708.85, 102209.85) | 23.07(20.68, 25.25) | -1.24(-1.42, -1.06) | <0.001 |
| South-East Asia Region | 6622(5608, 7283) | 1.25(1.06, 1.38) | 7567(6605, 8633) | 0.89(0.78, 1.02) | -1.19(-1.47 , -0.9) | <0.001 | 68321.68(60871.40, 75570.01) | 24.60(21.91, 27.21) | 113844.46(99544.38, 128885.54) | 17.92(15.67, 20.28) | -1.10(-1.31, -0.88) | <0.001 |
| Western Pacific Region | 17385(15538, 19387) | 2.48(2.21, 2.76) | 9902(8691, 11295) | 1.45(1.28, 1.66) | -1.88(-2.56 , -1.21) | <0.001 | 373089.40(331507.42, 416052.21) | 87.62(77.85, 97.71) | 498610.04(422872.54, 576746.63) | 54.33(46.08, 62.85) | -1.66(-1.81, -1.51) | <0.001 |
| **GBD regions** | | | | | | | | | | | | |
| Andean Latin America | 364(323, 408) | 2.36(2.09, 2.64) | 457(351, 581) | 1.78(1.37, 2.26) | -0.96(-1.55 , -0.35) | 0.002 | 5800.51(5058.00, 6577.95) | 75.62(65.94, 85.75) | 11152.70(8733.65, 13913.98) | 56.37(44.14, 70.32) | -1.14(-1.67, -0.60) | <0.001 |
| Australasia | 39(36, 43) | 0.48(0.44, 0.52) | 28(24, 33) | 0.29(0.25, 0.34) | -1.72(-2.16 , -1.27) | <0.001 | 1707.82(1561.10, 1842.52) | 22.69(20.74, 24.48) | 1959.69(1620.64, 2312.21) | 14.19(11.74, 16.75) | -1.61(-1.83, -1.40) | <0.001 |
| Caribbean | 134(106, 153) | 0.90(0.72, 1.03) | 152(117, 187) | 0.84(0.65, 1.03) | -0.22(-0.52 , 0.09) | 0.167 | 2787.50(2476.90, 3085.95) | 30.91(27.47, 34.22) | 3899.98(3234.34, 4620.41) | 22.54(18.70, 26.71) | -1.00(-1.33, -0.67) | <0.001 |
| Central Asia | 824(786, 869) | 2.89(2.76, 3.05) | 594(529, 672) | 1.57(1.40, 1.77) | -2.09(-2.66 , -1.52) | <0.001 | 12316.79(11606.05, 13000.50) | 77.88(73.38, 82.20) | 11030.39(9721.17, 12494.48) | 38.39(33.84, 43.49) | -2.42(-2.57, -2.26) | <0.001 |
| Central Europe | 646(623, 671) | 1.40(1.35, 1.46) | 249(216, 284) | 0.70(0.61, 0.80) | -2.43(-2.7 , -2.16) | <0.001 | 25634.78(24482.28, 26625.44) | 53.43(51.03, 55.50) | 19724.98(17032.91, 22441.26) | 32.42(27.99, 36.88) | -1.74(-1.97, -1.51) | <0.001 |
| Central Latin America | 914(881, 947) | 1.34(1.29, 1.39) | 1227(1024, 1457) | 1.22(1.01, 1.44) | -0.26(-0.56 , 0.04) | 0.094 | 14447.55(13569.49, 15149.52) | 45.45(42.68, 47.65) | 25670.64(21467.96, 30480.25) | 30.76(25.72, 36.52) | -1.38(-1.71, -1.05) | <0.001 |
| Central Sub-Saharan Africa | 186(136, 237) | 0.90(0.65, 1.14) | 279(204, 365) | 0.54(0.39, 0.70) | -1.76(-1.88 , -1.64) | <0.001 | 2565.41(1989.77, 3248.82) | 28.75(22.30, 36.41) | 3992.36(2982.93, 5257.93) | 17.60(13.15, 23.18) | -1.67(-1.83, -1.51) | <0.001 |
| East Asia | 14355(12466, 16300) | 2.53(2.20, 2.87) | 8749(7519, 10098) | 1.70(1.46, 1.96) | -1.43(-2.16 , -0.69) | <0.001 | 298506.66(258489.02, 340455.70) | 92.37(79.99, 105.36) | 422826.89(352983.37, 497569.26) | 58.46(48.80, 68.79) | -1.62(-1.85, -1.40) | <0.001 |
| Eastern Europe | 2513(2330, 2627) | 2.93(2.72, 3.06) | 1136(1002, 1280) | 1.66(1.46, 1.87) | -1.66(-2.4 , -0.92) | <0.001 | 78313.30(74779.75, 81013.83) | 87.80(83.84, 90.83) | 43671.53(38587.91, 48886.95) | 41.94(37.06, 46.95) | -2.42(-3.23, -1.59) | <0.001 |
| Eastern Sub-Saharan Africa | 671(492, 793) | 0.95(0.70, 1.13) | 923(748, 1155) | 0.55(0.45, 0.69) | -1.85(-1.93 , -1.77) | <0.001 | 7657.67(6534.08, 8757.53) | 26.14(22.31, 29.90) | 11126.59(9569.03, 12942.38) | 16.25(13.98, 18.91) | -1.63(-1.74, -1.52) | <0.001 |
| High-income Asia Pacific | 2514(2411, 2596) | 3.72(3.57, 3.84) | 518(480, 559) | 0.99(0.91, 1.06) | -4.47(-4.81 , -4.13) | <0.001 | 66863.44(63371.99, 69429.54) | 94.62(89.68, 98.25) | 65683.18(55934.88, 72098.10) | 59.35(50.54, 65.15) | -1.61(-1.87, -1.35) | <0.001 |
| High-income North America | 448(433, 464) | 0.40(0.38, 0.41) | 376(353, 405) | 0.31(0.29, 0.33) | -0.84(-1.3 , -0.38) | <0.001 | 20665.36(19391.88, 21482.98) | 19.47(18.27, 20.24) | 21193.99(19346.26, 22647.65) | 12.05(11.00, 12.88) | -1.65(-1.86, -1.44) | <0.001 |
| North Africa and Middle East | 1549(1308, 1775) | 1.14(0.96, 1.31) | 1996(1658, 2398) | 0.77(0.64, 0.93) | -1.32(-1.4 , -1.23) | <0.001 | 38430.18(36217.85, 40094.51) | 46.26(43.59, 48.26) | 63077.54(55127.22, 71005.55) | 30.75(26.87, 34.61) | -1.52(-1.60, -1.44) | <0.001 |
| Oceania | 48(37, 61) | 1.84(1.41, 2.30) | 107(79, 143) | 1.96(1.44, 2.62) | 0.18(0.08 , 0.29) | 0.001 | 21931.98(18499.48, 24873.00) | 33.49(28.25, 37.98) | 37378.20(32687.69, 42096.59) | 21.47(18.77, 24.18) | -0.39(-0.53, -0.26) | <0.001 |
| South Asia | 5190(4459, 5789) | 1.20(1.03, 1.34) | 6981(6075, 8025) | 0.91(0.79, 1.04) | -0.99(-1.32 , -0.66) | <0.001 | 361.02(272.56, 467.89) | 29.85(22.54, 38.69) | 799.40(579.29, 1079.50) | 26.62(19.29, 35.94) | -0.88(-1.27, -0.50) | <0.001 |
| Southeast Asia | 2103(1730, 2370) | 1.07(0.88, 1.20) | 1643(1416, 1895) | 0.60(0.52, 0.70) | -1.93(-2.04 , -1.81) | <0.001 | 52028.40(45649.65, 58340.20) | 22.96(20.14, 25.74) | 91882.73(78921.20, 106302.19) | 17.69(15.20, 20.47) | -1.84(-1.95, -1.73) | <0.001 |
| Southern Latin America | 215(202, 228) | 1.13(1.06, 1.19) | 191(165, 217) | 0.75(0.65, 0.85) | -1.39(-1.68 , -1.1) | <0.001 | 26025.37(22305.25, 29420.84) | 26.64(22.83, 30.12) | 36330.81(31479.25, 41499.92) | 15.58(13.50, 17.80) | -1.28(-1.51, -1.03) | <0.001 |
| Southern Sub-Saharan Africa | 210(190, 230) | 0.96(0.86, 1.05) | 203(162, 251) | 0.60(0.48, 0.75) | -1.54(-2.27 , -0.82) | <0.001 | 8261.29(7632.60, 8896.38) | 53.26(49.21, 57.35) | 9616.46(8336.49, 10977.92) | 36.50(31.64, 41.67) | -1.07(-1.49, -0.65) | 0.001 |
| Tropical Latin America | 757(723, 794) | 1.18(1.12, 1.23) | 773(726, 819) | 0.87(0.81, 0.92) | -1.07(-1.34 , -0.8) | <0.001 | 2230.86(1953.65, 2522.42) | 21.91(19.18, 24.77) | 3438.45(3020.19, 3900.96) | 16.17(14.20, 18.34) | -1.75(-1.93, -1.57) | <0.001 |
| Western Europe | 1142(1110, 1174) | 0.79(0.77, 0.82) | 497(461, 533) | 0.38(0.35, 0.41) | -2.53(-2.7 , -2.37) | <0.001 | 15394.62(14384.48, 16273.55) | 44.49(41.57, 47.03) | 22354.22(20303.98, 24140.61) | 26.43(24.00, 28.54) | -2.06(-2.21, -1.90) | <0.001 |
| Western Sub-Saharan Africa | 449(381, 521) | 0.63(0.54, 0.73) | 815(657, 1001) | 0.45(0.37, 0.56) | -1.12(-1.24 , -1.01) | <0.001 | 78943.99(74726.33, 81850.30) | 46.65(44.16, 48.37) | 60547.74(53866.63, 65487.94) | 25.72(22.89, 27.82) | -0.99(-1.04, -0.93) | <0.001 |
| **SDI level** | | | | | | | | | | | | |
| High SDI | 4100(3986,4198) | 1.27(1.23,1.30) | 1555(1479,1641) | 0.47(0.45,0.50) | -3.4(-3.58 , -3.22) | <0.001 | 149452.10(142075.69, 154104.33) | 45.89(43.62, 47.31) | 137231.23(121189.97, 147536.12) | 26.58(23.47, 28.58) | -1.87(-1.98, -1.76) | <0.001 |
| High-middle SDI | 9603(8910,10413) | 1.99(1.84,2.15) | 6039(5484,6638) | 1.17(1.06,1.28) | -1.77(-2.21 , -1.33) | <0.001 | 266304.33(247491.78, 284543.46) | 73.33(68.15, 78.35) | 272874.79(240194.96, 304235.23) | 40.88(35.99, 45.58) | -2.00(-2.21, -1.79) | <0.001 |
| Low SDI | 2014(1620,2300) | 1.04(0.84,1.19) | 3160(2715,3671) | 0.71(0.61,0.82) | -1.30(-1.44 , -1.2) | <0.001 | 25102.24(21913.84, 28183.26) | 27.20(23.75, 30.54) | 40600.16(35780.16, 45784.87) | 19.52(17.20, 22.01) | -1.14(-1.19, -1.08) | <0.001 |
| Low-middle SDI | 6419(5606,7038) | 1.44(1.26,1.58) | 7714(6909,8561) | 1.05(0.94,1.16) | -1.0(-1.3 , -0.86) | <0.001 | 78947.62(71255.31, 86552.49) | 34.20(30.87, 37.49) | 133724.07(120062.60, 147451.59) | 26.49(23.79, 29.21) | -0.88(-0.98, -0.77) | <0.001 |
| Middle SDI | 13123(11819,14426) | 1.76(1.58,1.93) | 9412(8581,10351) | 1.01(0.92,1.11) | -1.84(-2.37 , -1.31) | <0.001 | 230852.95(205796.03, 257591.83) | 59.40(52.95, 66.28) | 334315.32(291065.59, 377693.45) | 36.85(32.09, 41.64) | -1.66(-1.81, -1.52) | <0.001 |

UI: uncertainty interval; CI: confidence interval; AAPC: average annual percentage change; SDI: socio-demographic index; Data in parentheses are 95% uncertainty intervals for rate and cases for mortality, incidence, and 95% Cis for AAPCs.

**Supplementary Table 3** List of countries with significantly increased average annual percentage changes of mortality rate

| **Country** | **GBD-region** | **GBD-Super regions** | **WHO** | **AAPC(95% Confidence Interval)** | **P value** |
| --- | --- | --- | --- | --- | --- |
| **Young-onset** | | | | | |
| Eswatini | Southern Sub‐Saharan Africa | Sub‐Saharan Africa | African Region | 0.63(0.19 , 1.08) | 0.005 |
| Kenya | Eastern Sub‐Saharan Africa | Eastern Sub‐Saharan Africa | African Region | 0.34(0.21 , 0.47) | <0.001 |
| Lesotho | Southern Sub‐Saharan Africa | Southern Sub‐Saharan Africa | African Region | 1.89(1.59 , 2.19) | <0.001 |
| Zimbabwe | Southern Sub‐Saharan Africa | Southern Sub‐Saharan Africa | African Region | 1.62(0.93 , 2.33) | <0.001 |
| Pakistan | Pakistan | South Asia | Eastern Mediterranean Region | 0.49(0.31 , 0.68) | <0.001 |
| Belize | Caribbean | Latin America and Caribbean | Region of the Americas | 0.96(0.13 , 1.81) | 0.024 |
| Dominican Republic | Caribbean | Latin America and Caribbean | Region of the Americas | 0.56(0.11 , 1.02) | 0.015 |
| Guam | Oceania | Southeast Asia, East Asia, and Oceania | Western Pacific Region | 1.23(0.44 , 2.03) | 0.002 |
| Marshall Islands | Oceania | Southeast Asia, East Asia, and Oceania | Western Pacific Region | 0.59(0.47 , 0.7) | <0.001 |
| Solomon Islands | Oceania | Southeast Asia, East Asia, and Oceania | Western Pacific Region | 0.52(0.33 , 0.71) | <0.001 |
| Tonga | Oceania | Southeast Asia, East Asia, and Oceania | Western Pacific Region | 0.34(0.10 , 0.59) | 0.006 |
| Vanuatu | Oceania | Southeast Asia, East Asia, and Oceania | Western Pacific Region | 0.43(0.15 , 0.71) | 0.003 |
| **Late-onset** | | | | | |
| Honduras | Region of the Americas | Central Latin America | Latin America and Caribbean | 0.64(0.33, 0.96) | <0.001 |
| Lesotho | African Region | Southern Sub‐Saharan Africa | Sub‐Saharan Africa | 0.27(0.12, 0.42) | <0.001 |
| United States Virgin Islands | Region of the Americas | High‐income North America | High‐income | 0.98(0.71, 1.25) | <0.001 |


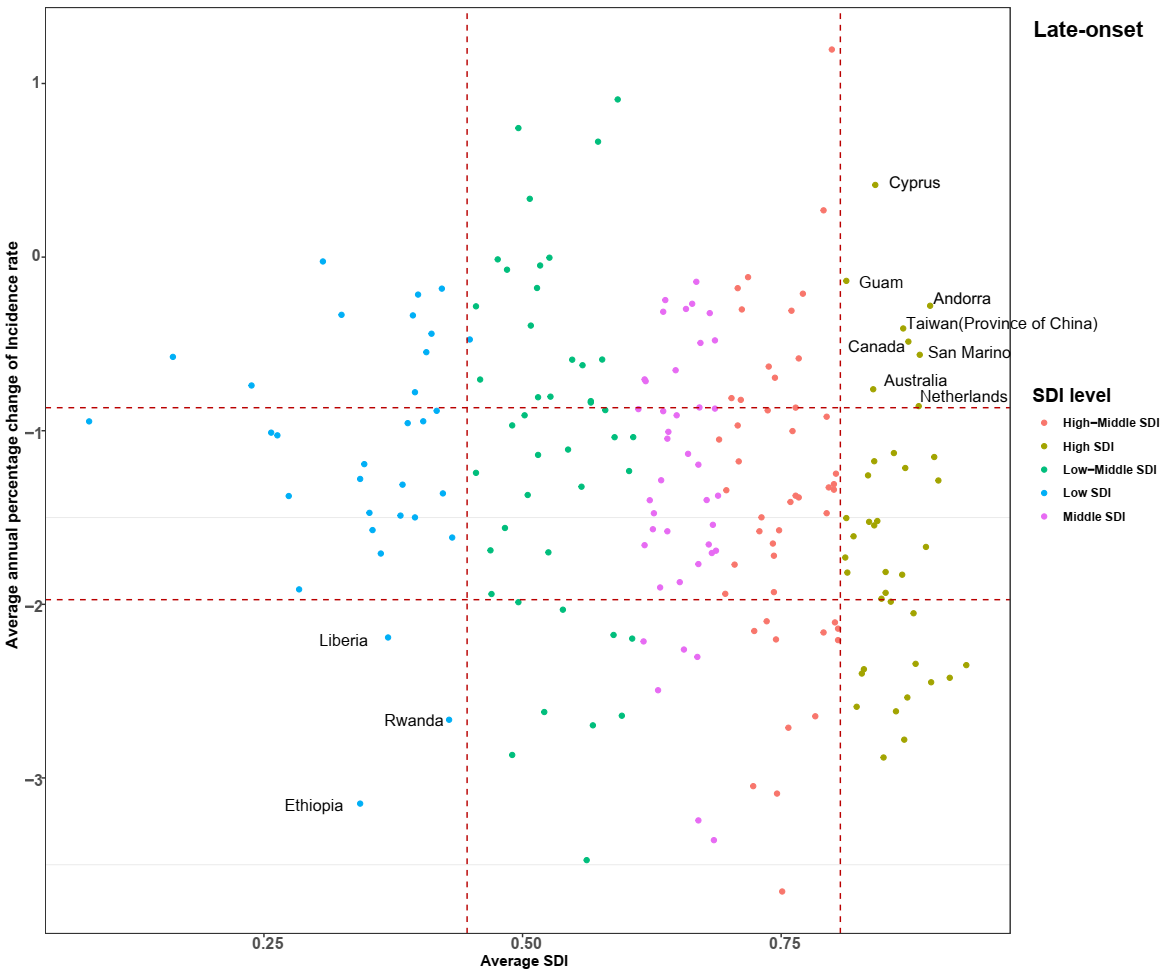

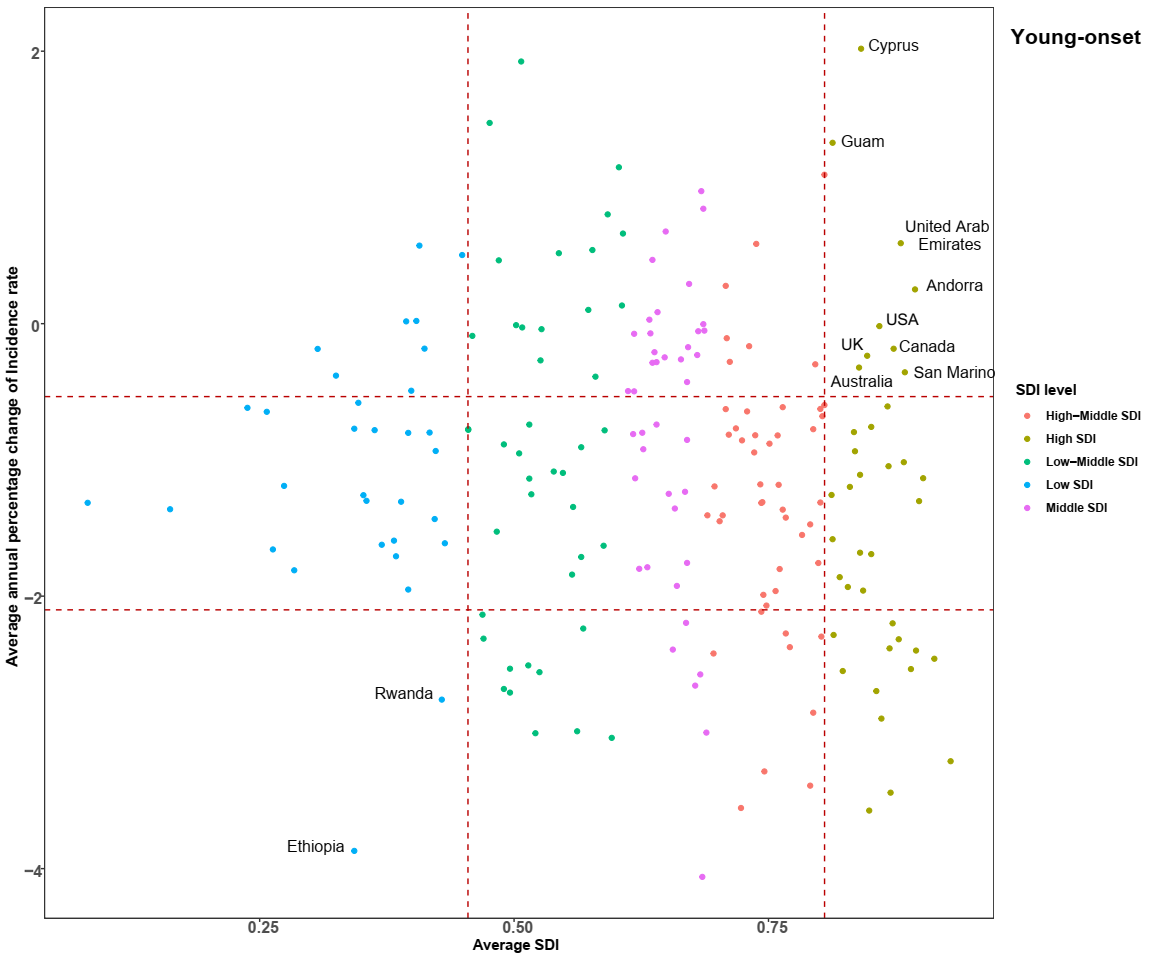
**Supplementary Figure 1** Countries classification based on AAPC level (25 and 75 percentile) and SDI level


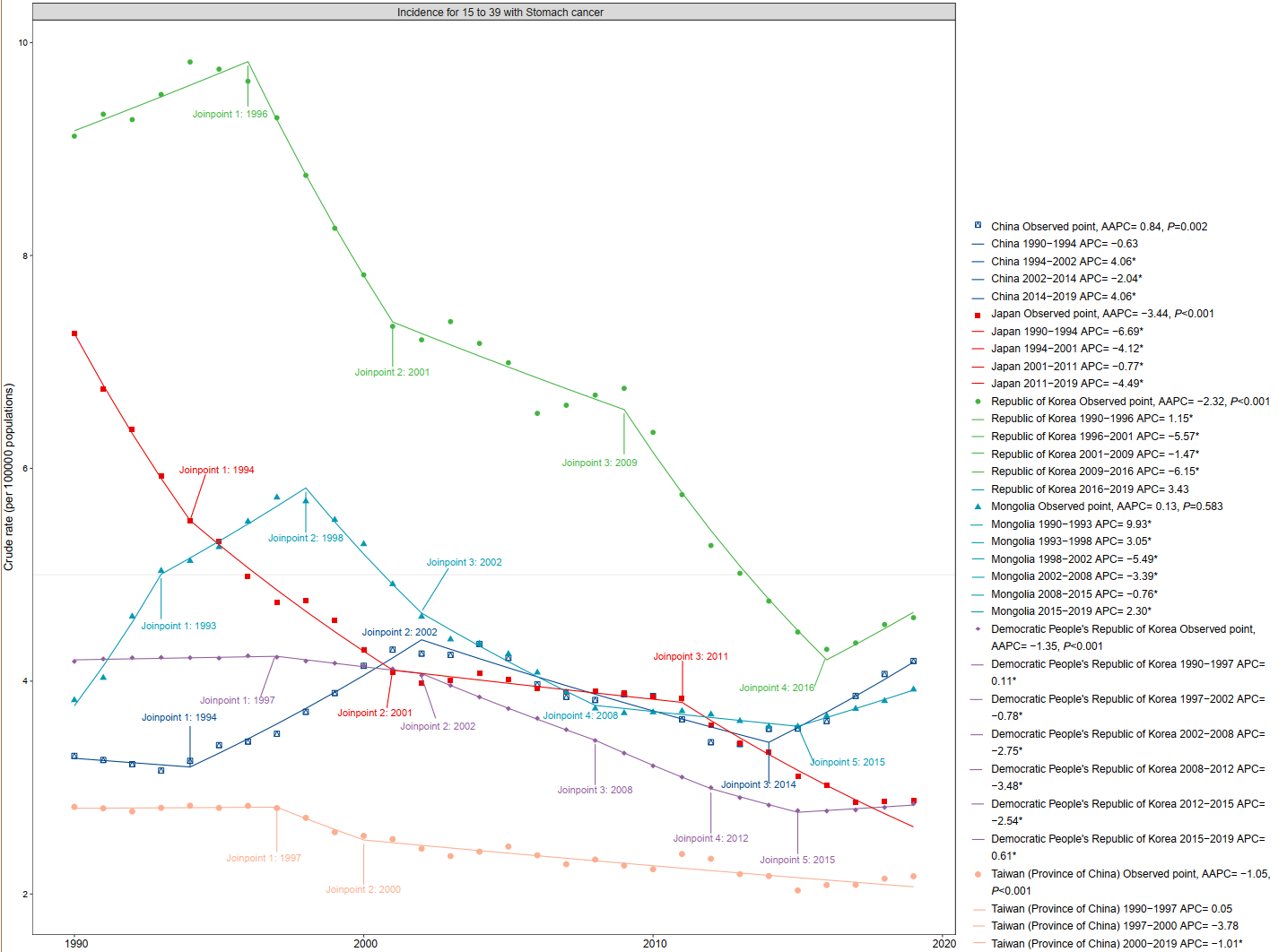
**Supplementary Figure 2** Joinpoint regression result of incidence rate in East Asia(UNSD classification)


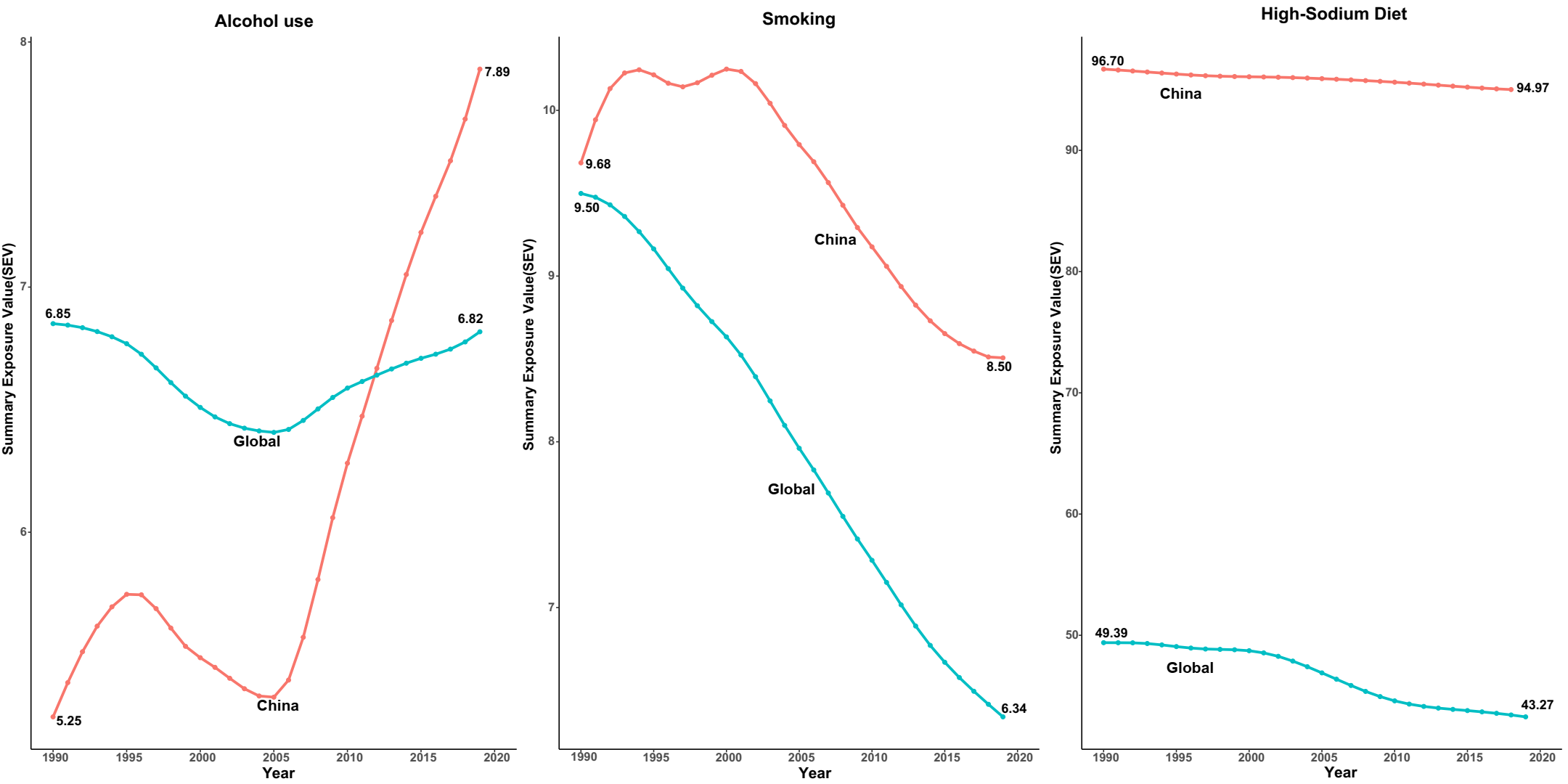
**Supplementary Figure 3** Summary Exposure Value of alcohol use, smoking, and high-sodium diet of global and China among young individuals

**Supplementary Figure 4A** Joinpoint regression result of Japan and the Republic of Korea(late-onset)


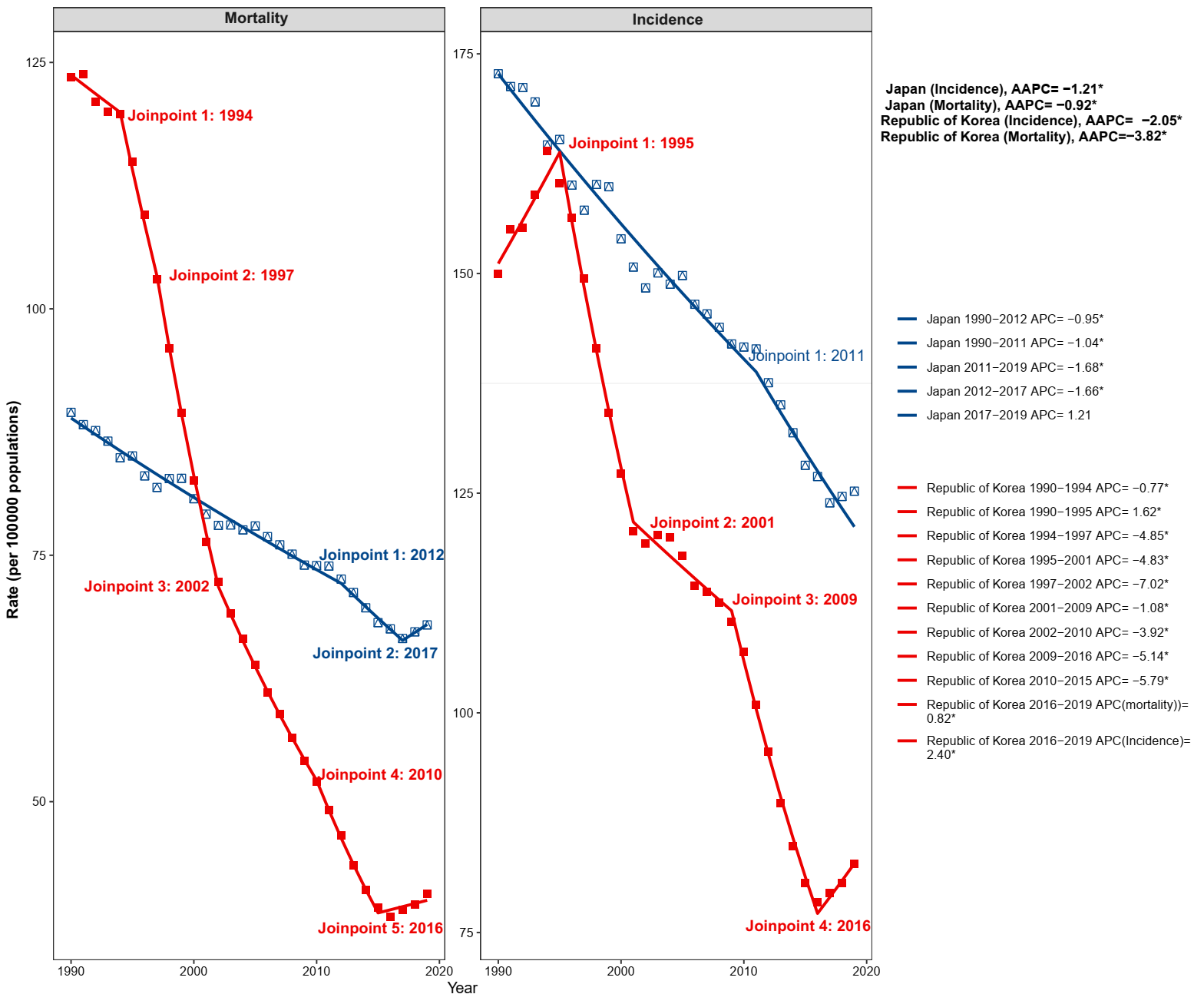


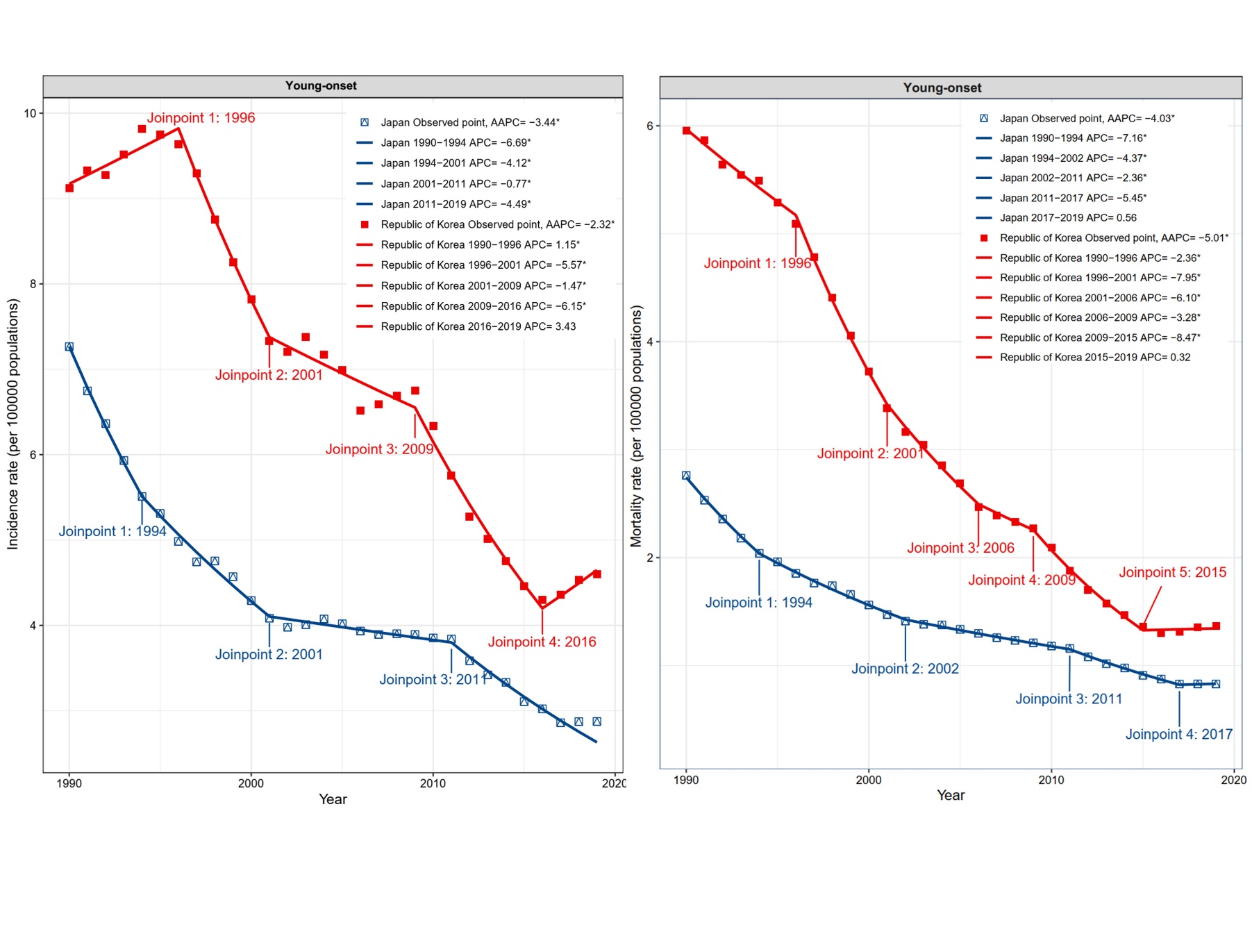
**Supplementary Figure 4B** Joinpoint regression result of Japan and the Republic of Korea(young-onset)


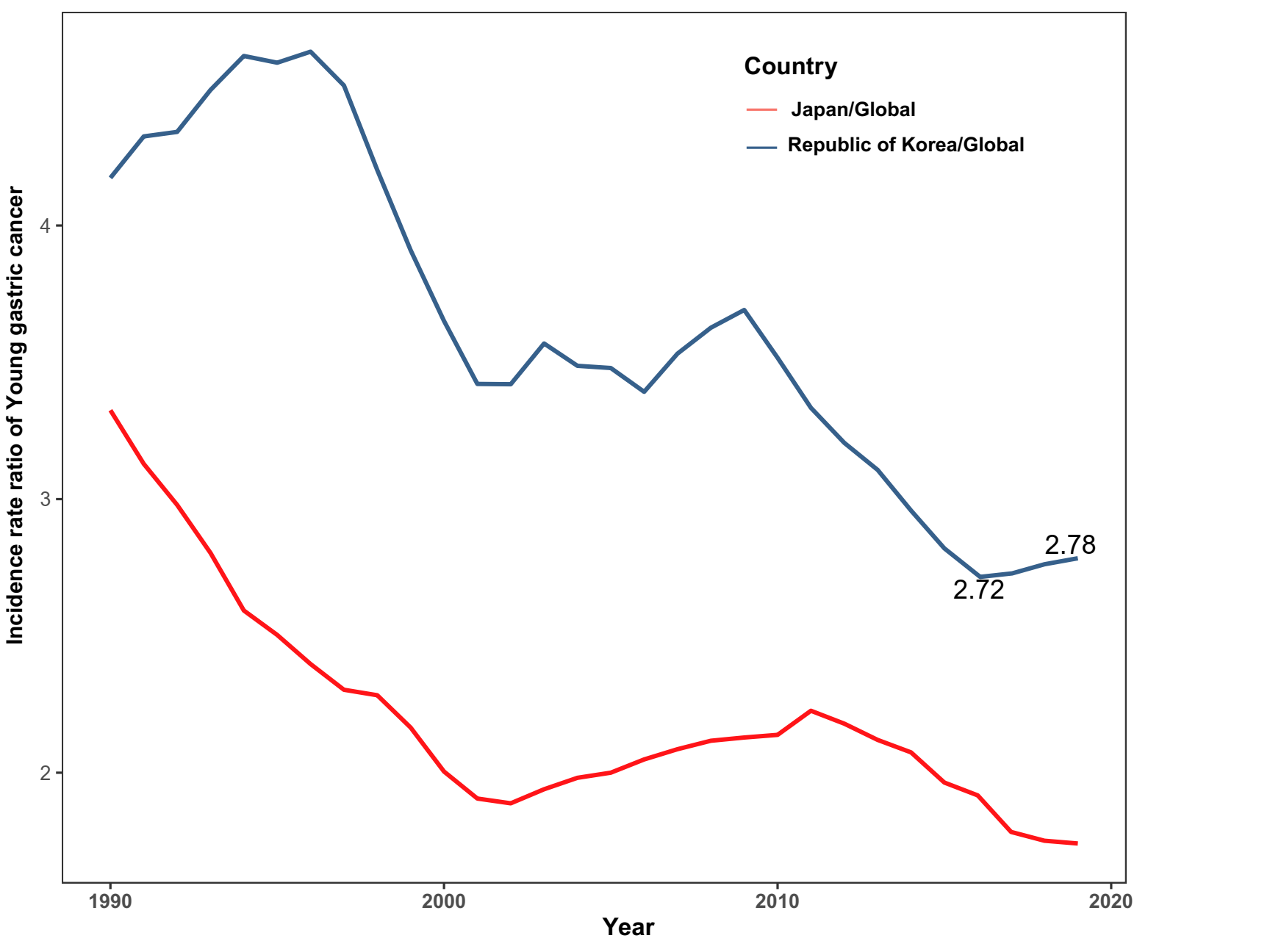
**Supplementary Figure 5** Temporal trend of young-onset gastric cancer incidence rate ratio between the Republic of Korea & Japan versus the global level


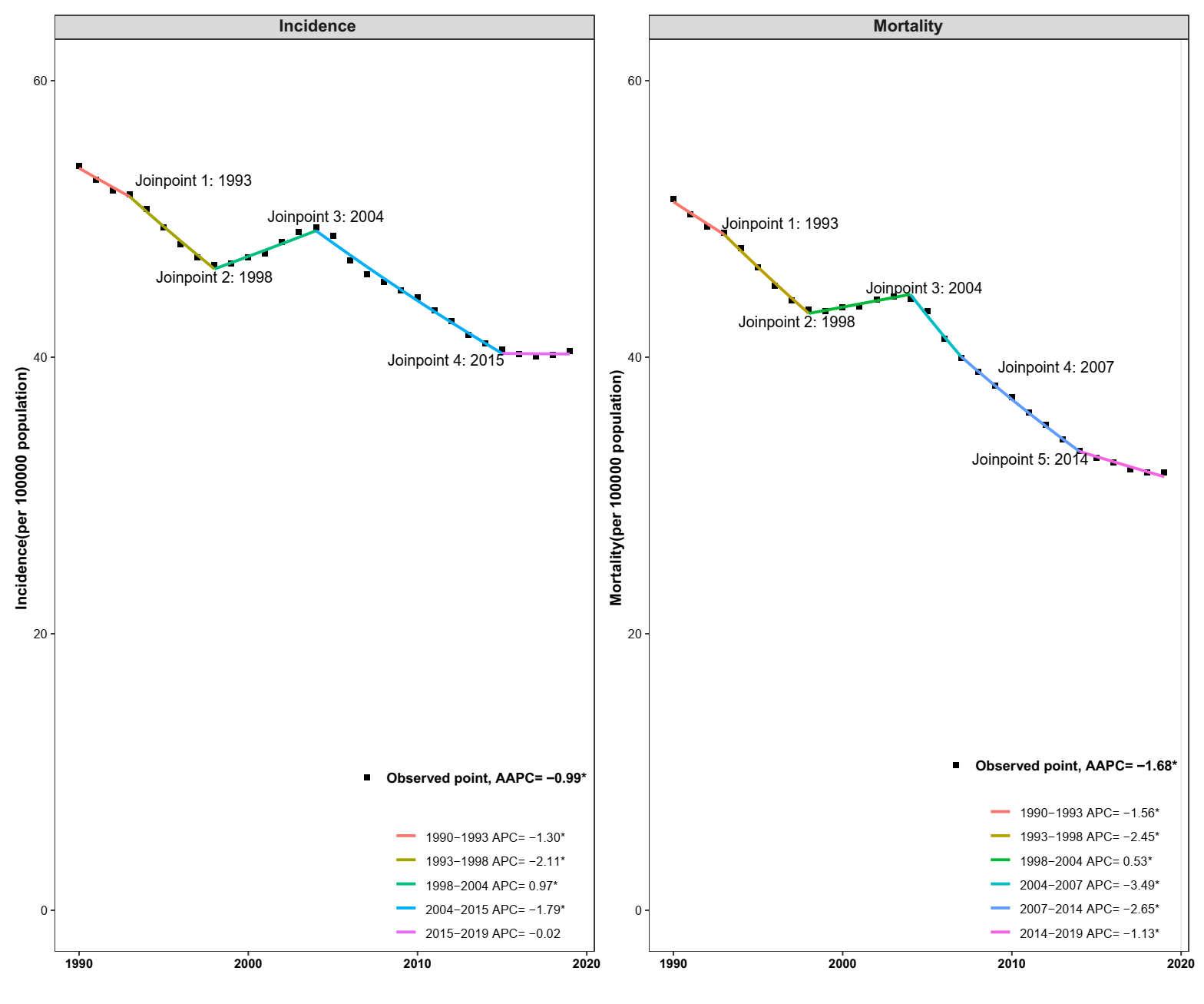
**Supplementary Figure 6** Joinpoint regression result of global data excluding Japan and the Republic of Korea


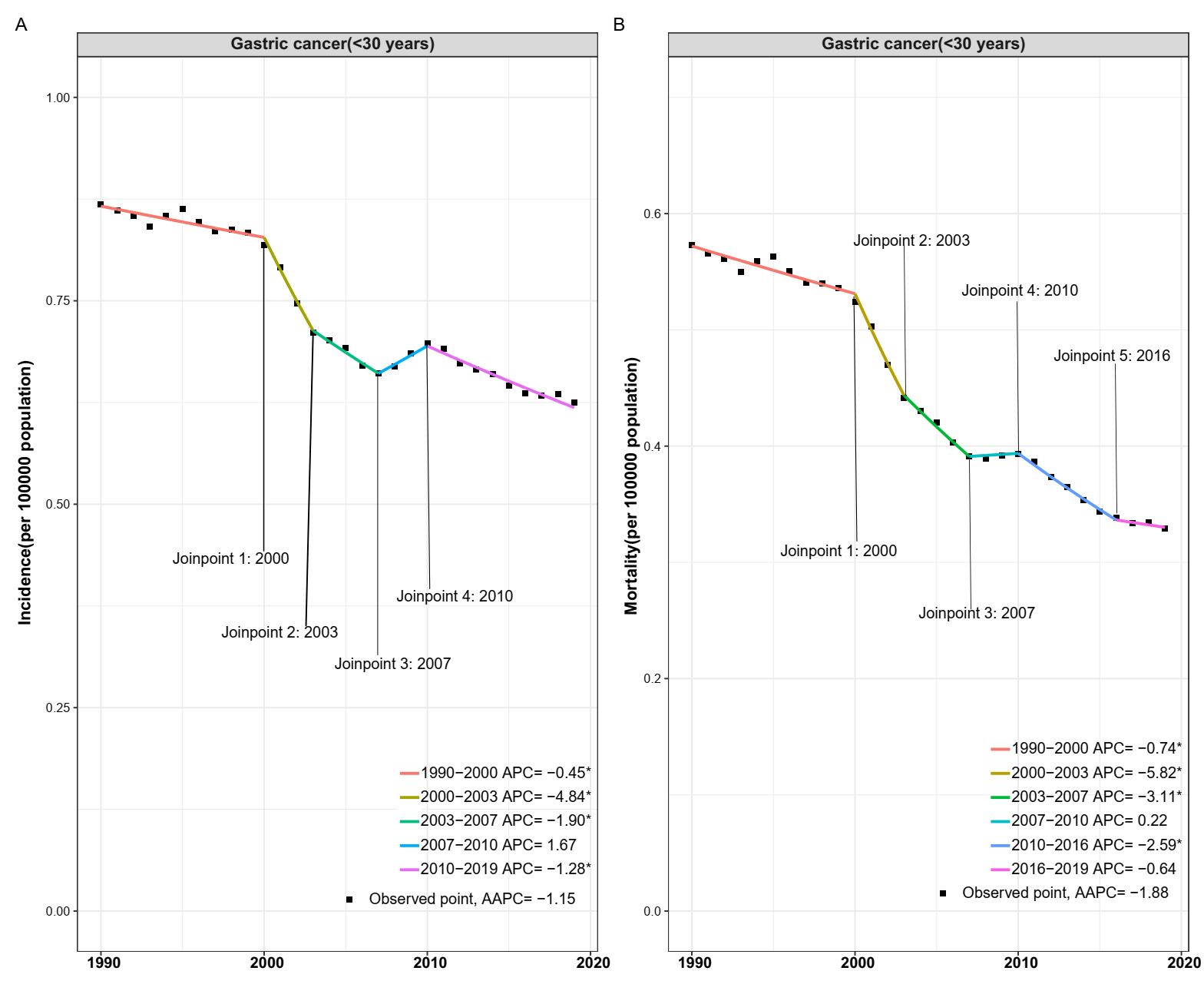
**Supplementary Figure 7** Joinpoint regression result of young gastric cancer incidence (A) and Mortality(B) which set the cut-off age at 30 years


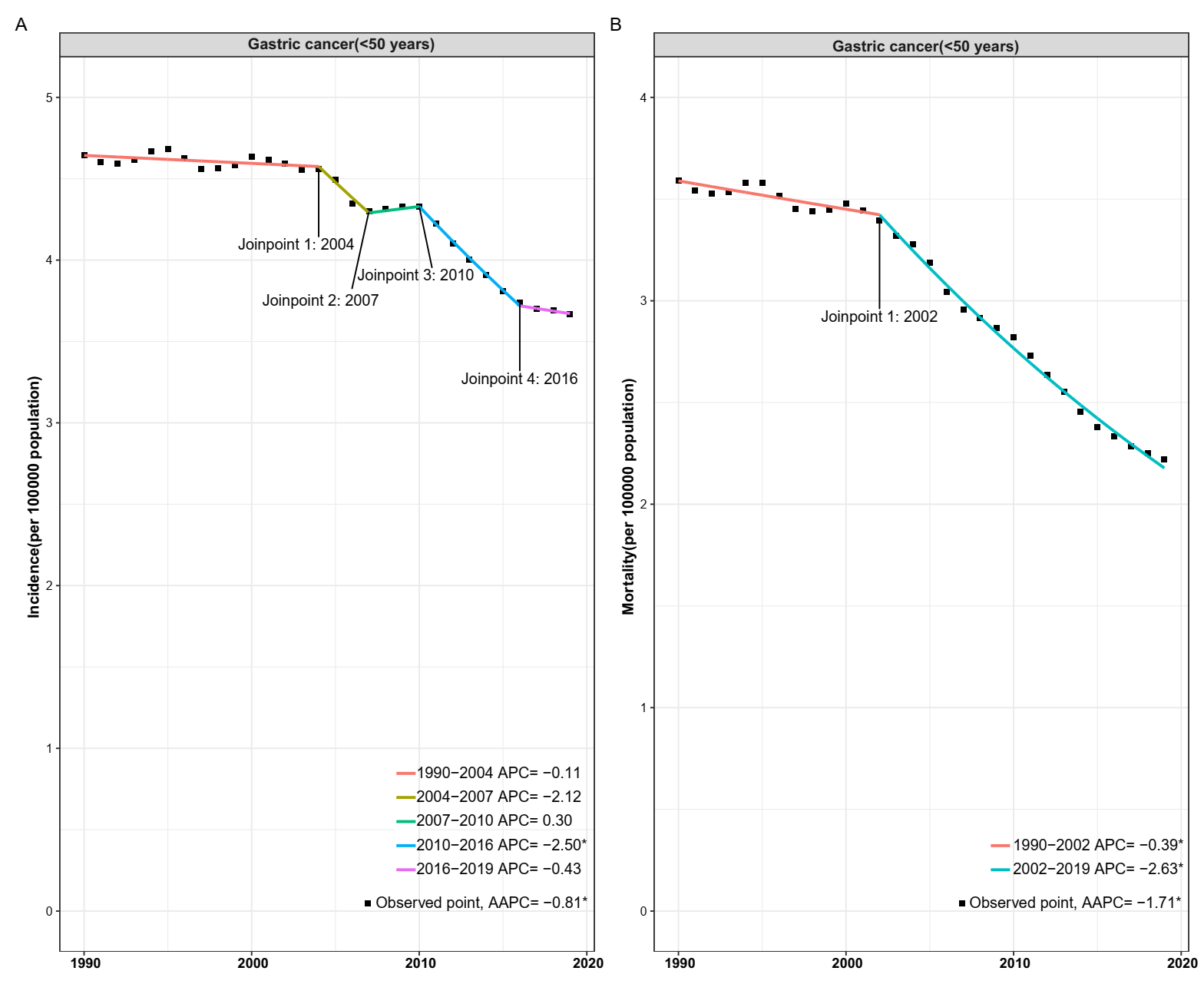
**Supplementary Figure 8** Joinpoint regression result of young gastric cancer incidence (A) and Mortality(B) which set the cut-off age at 50 years
